# Analysis of Pharmacological Properties of *Nigella sativa* L. Bioactive Compounds and Their Therapeutic Relevance in the Management of Type 2 Diabetes Mellitus

**DOI:** 10.3390/life15111681

**Published:** 2025-10-29

**Authors:** Monica Tabita Morar (Romocea), Annamaria Pallag, Cristina Burlou-Nagy (Fati), Laura Grațiela Vicaș, Ioana Lavinia Dejeu, Tünde Horvath, Diana Bei, Cosmin Vesa

**Affiliations:** 1 Doctoral School of Biological and Biomedical Sciences, University of Oradea, 410087 Oradea, Romania; morar.monicatabita@student.uoradea.ro (M.T.M.); apallag@uoradea.ro (A.P.); 2 Department of Pharmacy, Faculty of Medicine and Pharmacy, University of Oradea, 410028 Oradea, Romania; lvicas@uoradea.ro (L.G.V.); ioana.dejeu@didactic.uoradea.ro (I.L.D.); thorvath@uoradea.ro (T.H.); 3 Department of Medical Disciplines, Faculty of Medicine and Pharmacy, University of Oradea, 410028 Oradea, Romania; diana.bei@didactic.uoradea.ro; 4 Department of Preclinical Disciplines, Faculty of Medicine and Pharmacy, University of Oradea, 410028 Oradea, Romania; cosmin.vesa@csd.uoradea.ro

**Keywords:** *Nigella sativa* L., bioactive compounds, thymoquinone, diabetes mellitus, antioxidant, anti-inflammatory effect, antidiabetic, glycemia, clinical studies

## Abstract

*Nigella sativa* L. is a species of the Ranunculaceae family and belongs to the genus Nigella, which comprises 14 species native to the regions of the Eastern Mediterranean, North Africa, and subcontinental India. Several significant groups of bioactive compounds, with pharmacological activities, have been isolated from the *Nigella sativa* L. species. Numerous beneficial effects have been demonstrated for these compounds. This review provides a comprehensive summary of the chemical constituents, bioactive compounds, modern administration methods, studies on the antidiabetic potential, evidence in commonly associated type 2 diabetes mellitus (T2DM) conditions, and evidence of T2DM complications. Research shows that *Nigella sativa* L. can be successfully included in complementary and alternative therapy for T2DM pathology, having multiple benefits both in diseases associated with and in complications of T2DM.

## 1. Introduction

Herbal medicine, known as phytotherapy, plays a significant role in the management and treatment of various diseases around the world [1]. Diabetes is one of the most critical public health challenges globally, affecting millions of people and imposing substantial economic and social costs [2,3]. In the context in which the diabetic population is continuously growing, the search for complementary and alternative treatments, which are both effective and less associated with the adverse effects of conventional medication, has become a priority [4,5,6].

Diabetes mellitus is a complex metabolic disorder characterized by disturbances in the metabolism of carbohydrates, fats, and proteins that can lead to chronic hyperglycemia. This disease may include defects in insulin secretion and/or action [7]. The most common form of diabetes is type 2 diabetes, which accounts for about 90% of diabetes cases. Type 2 diabetes mellitus (T2DM) is mainly due to the failure of tissues to respond to insulin or synthesize enough insulin [8].

Data from the literature reveal that diabetes affects human quality of life by causing major risk factors in terms of the possibility of complications such as stroke, kidney failure, and diabetic retinopathy, which is a major cause of blindness, leading to significant morbidity and premature mortality [9,10,11]. This phenomenon poses serious problems regarding multiple complications, such as cardiovascular system damage, polyneuropathy, retinopathy, chronic kidney disease, and dyslipidemia, thus complicating the clinical management of the disease [12]. The pathology of type 2 diabetes mellitus (T2DM) is a significant public health problem globally, affecting millions of people and being associated with an increased risk of cardiovascular complications, stroke, and other metabolic diseases [9,13,14].

The complications of diabetes are varied and affect multiple organs and systems: damage to the cardiovascular system—diabetes increases the risk of atherosclerosis, heart attack myocardial and stroke [13]; polyneuropathy—damage to the peripheral nerves causes loss of sensation and can cause chronic damage, especially in the lower limbs [9]; retinopathy—damage to the ocular blood vessels can lead to vision loss in the advanced stage of the disease [9]; chronic kidney disease—diabetes is the leading cause of kidney failure due to deterioration of glomerular function [15,16]; and dyslipidemia—lipid imbalances, common in diabetic people, contribute to increasing cardiovascular risk [17]. These complications underline the need for therapeutic strategies that address not only hyperglycemia but also the systemic manifestations of the disease, thus contributing to improving patients’ quality of life [14].

Conventional treatment of diabetes involves the administration of insulin and oral hypoglycemic drugs, such as sulfonylureas, biguanides, and glinides [8]. Although these therapies have revolutionized diabetes management, they still have adverse effects [18].

The adverse effects of classic medication in diabetes can be hypoglycemia and hyperglycemia—sudden fluctuations in blood glucose levels can cause severe complications [19]; systemic side effects—long-term administration of these classes of drugs can cause nausea, gastrointestinal disorders and other side effects in the body [20]; and insulin resistance—in some cases, patients may develop insulin tolerance, thus complicating long-term treatment [21]. Therefore, the identification and implementation of complementary therapeutic options, such as the integration of medicinal plants with hypoglycemic effects, becomes essential for reducing side effects and optimizing glycemic control [18,22].

Treatment strategies for diabetes have improved in recent decades. However, antidiabetic drugs can have serious side effects such as hypoglycemic coma, liver and kidney disorders, or urinary tract infections [16,20]. Thus, it is recommended to use medicinal plants as an adjuvant therapy that is complementary in the management of diabetes mellitus [7,19,23,24,25]. Recent pharmacological studies have revealed antidiabetic properties of medicinal plants, antilipids, and hypoglycemic drugs [19,26,27]. The major groups of phytochemicals are alkaloids, terpenes, carotenoids, essential oils, flavonoids, phytosterols, and polyphenols [11,15,18]. Recent studies have highlighted the potential of the *Nigella sativa* L. species (black seed) in T2DM management, due to its anti-inflammatory, antioxidant properties, and improved insulin sensitivity [13,14,17].

*Nigella sativa* L. is a species of the Ranunculaceae family and belongs to the genus Nigella comprising 14 species, including *N. arvensis*, *N. ciliaris*, *N. damascene*, *N. hispanica*, *N. integriflolia*, *N. nigellastrum*, *N. orientalis*, and *N. sativa*; it is also known as black, black seeds or “black cumin”, having a long history of use in traditional medicine. It is an annual herbaceous plant that grows in countries bordering the Mediterranean Sea; the plant has a green color with finely divided linear leaves; the flowers are pale blue and white, with 5–10 petals; and the fruits are found in the form of swollen capsules, divided into 3–7 united follicles. Each follicle contains numerous black seeds, having an oval shape and a diameter of about 1 mm [28]. Native to the regions of the Eastern Mediterranean, North Africa, and subcontinental India, *Nigella sativa* L. (NS) is known for its diverse therapeutic properties and relative safety in use [29,30,31]. This plant has been used for thousands of years in traditional folk medicine as a protective and beneficial remedy for various ailments, black cumin seeds being mentioned in the Bible in the book of the prophet Isaiah [28,29,32]. *Nigella sativa* L. seeds have been used for thousands of years not only as a food ingredient, but also as a remedy for a wide range of ailments. In Islamic tradition, *Nigella sativa* L. seeds are considered “seeds of blessing” and are referred to as an almost universal remedy. Their historical use involves the inclusion in the therapy of respiratory diseases and inflammations, hypertension, and diabetes, demonstrating a medicinal potential that is particularly recognized in various cultures [33,34,35,36].

Regarding geographical distribution and biodiversity, NS is widely cultivated in the Mediterranean regions, North Africa, the Indian subcontinent, and in the areas of Southwest Asia. This wide geographical distribution made it possible to study various species and the chemotherapeutic variability of seeds, leading to the discovery of a wide range of active compounds [37,38]. NS is used in therapeutics in different forms: powders, capsules, essential oils, and various seed extracts [39,40].

**Figure 1:**
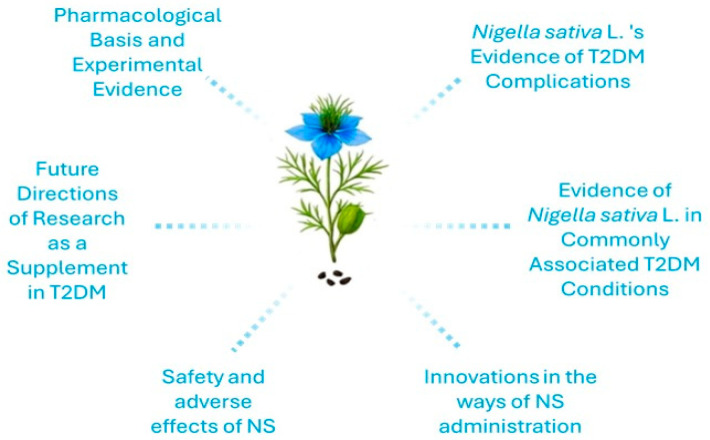
Presentation of the main research directions related to NS.

The purpose of this manuscript is to evaluate the biological effects of the active compounds of NS and their impact on the pathogenesis and evolution of DM, providing a relevant review of the literature and the main research directions related to NS, as shown in Figure 1.

To address recent advances, this review provides an updated synthesis that goes beyond previous analyses [32,33,41,42]. It integrates the latest preclinical and clinical findings from 2023–2025, offering a mechanistic perspective on the bioactive constituents of *Nigella sativa* in type 2 diabetes. Moreover, the review discusses innovative approaches such as nanotechnology-based formulations and targeted delivery systems, highlighting emerging strategies to enhance the antidiabetic potential of *N. sativa*. This updated perspective aims to bridge current knowledge gaps and guide future translational and therapeutic developments.

**Figure 2:**
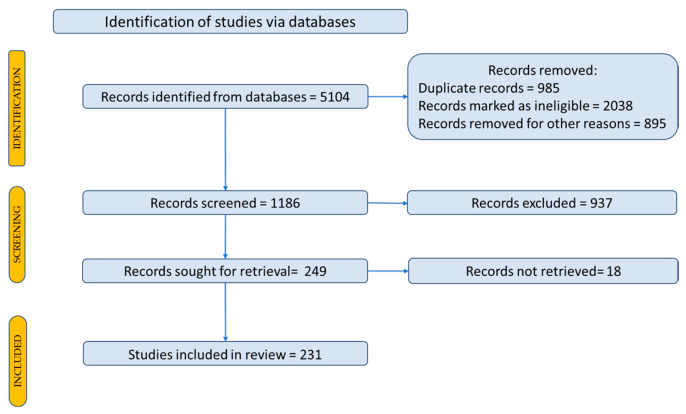
Prisma flow diagram for description of the selection process of the bibliographic sources.

To identify scientific articles published on *Nigella sativa* L., the articles were searched using representative keywords to locate the primary data, outcomes, and papers in the field. To search for articles, the following keywords were used: Nigella and *Nigella sativa*. A Prisma flow diagram was used to describe how to select the studies and which articles were included in the review, as shown in Figure 2 [41].

## 2. Pharmacological Basis and Experimental Evidence

One of the main reasons for the increased interest in NS is due to its complex chemical composition. NS seeds contain numerous compounds, most of which have relevant therapeutic effects. Among these compounds, thymoquinone (TQ) stands out as the most active phytoconstituent (the main constituent of volatile oil), but also other important constituents such as thymohydroquinone, dithymoquinone, thymol, carvacrol, p-cymen, α-thujene, γ-terpinene, limonene, citronellol, α-pinene, β-pinene, anethole, alkaloids, and saponins, contributing to the various therapeutic benefits of the plant [28,29,32]. This complex chemical profile is the foundation on which the multiple therapeutic effects observed in preclinical and clinical studies, including antidiabetic activity, are based [43,44,45]. NS stands out among the other *Nigella* spp. because the volatile oils from its seeds are very rich in TQ, thus constituting a promising candidate for both traditional and modern phytotherapy, presenting a considerable preventive and therapeutic, curative potential [46,47,48]. Studies have focused on NS seeds, which are the primary source of bioactive constituents of the plant. Their volatile oil contains mainly alkaloids (with two types of alkaloids: nigellimine or isoquinoline and nigellicin or pyrazole), terpenes, and phenolic compounds: quercetin, tocopherols, and phytosterols (β-sitosterol), salicylic acid [30,42,49].

The antidiabetic properties of NS (significant reduction in postprandial blood glucose, decrease in glycosylated hemoglobin A1c (HbA1c) values essential for long-term glycemic control, protection of pancreatic β cell function by improving insulin secretion) can be attributed mainly to the activity of the active compound thymoquinone and other phenolic compounds that contribute to multiple beneficial metabolic responses [50,51]. Recent studies have highlighted various mechanisms of action at the molecular level by which the bioactive compounds in NS, especially thymoquinones, influence glucose metabolism and reduce the effects of inflammation and oxidative stress, thus contributing to the relief of diabetes [14,49,52]. These mechanisms include: activation of the AMPK pathway and regulation of glucose metabolism, inhibition of digestive enzymes, and improvement of glucose absorption, improvement of the enzymatic activity of the endogenous antioxidant system with significant reduction in damage to insulin receptor cells, and protection of pancreatic β cells [49]. Although these mechanisms have been clearly demonstrated in preclinical cellular and animal models, translation to human studies has been inconsistent. In vitro and in vivo models typically use higher concentrations of thymoquinone (TQ) than those achievable through dietary supplementation or conventional dosing in humans. Moreover, while animal data consistently show improved glycemic control, insulin sensitivity, and β-cell preservation, clinical studies have only partially confirmed these effects, often reporting modest or variable improvements in fasting blood glucose and HbA1c. This gap may reflect differences in bioavailability, duration of exposure, and inter-individual variation in metabolism.

The activation of AMP protein kinase (AMPK), which presents itself as a metabolic sensor and regulates both catabolism and energy anabolism, is a relevant mechanism of action at the molecular level of TQ [52]. Activation of AMP-activated protein kinase (AMPK) is essential for regulating energy metabolism, and numerous studies in cellular and animal models have demonstrated that thymoquinones stimulate AMPK activity. This activation causes, among other things, an increase in GLUT4 levels in the muscles, thus facilitating glucose absorption and improving glycemic control. Activation of AMPK leads to increased translocation of the GLUT4 receptor (an insulin-sensitive glucose transporter) into muscle cells, favoring glucose utilization and lowering plasma glucose concentration. This mechanism has been confirmed in preclinical studies demonstrating the beneficial effects of NS on hyperglycemia and insulin resistance [14,52,53].

Inhibition of digestive enzymes and improvement of glucose absorption are other molecular mechanisms of the antidiabetic activity of NS [18]. Some studies have shown that the phytoconstituents in NS can inhibit digestive enzymes involved in carbohydrate absorption through the inhibitory action of alpha-amylase and alpha-glucosidase, leading to a reduced increase in postprandial blood glucose [54,55,56,57,58,59]. This synergistic action of bioactive compounds, often combined with other plants such as Cuminum cyminum (cumin), supports the idea of using combination preparations in T2DM management [14].

Another relevant mechanism in the action of NS compounds is the ability to activate the sirtuin 1 protein (SIRT1) and reduce oxidative stress. TQ, by inhibiting the NADPH oxidase complex, decreases the production of reactive oxygen species (ROS) in inflammation-activated cells. This reduction in oxidative stress is essential in preventing cell damage and the progression of diabetic complications. Excessive oxidation and oxidative stress play a significant role in the pathogenesis of T2DM, with studies suggesting that NS can reduce levels of oxidative stress due to free radicals and improve the enzymatic activity of the endogenous antioxidant system [49,60]. This action significantly reduces damage to insulin receptor cells and protects pancreatic β cells, improving insulin secretion. NS has strong antioxidant properties, so the close correspondence of the pathology of DM with oxidative stress determines the effectiveness of thymoquinone supplementation, which supports the endogenous arsenal of cellular antioxidant enzymes and prevents endothelial dysfunction [49,61,62]. However, clinical evidence for these antioxidants and cytoprotective effects remains limited. Only a few randomized controlled trials have evaluated oxidative stress biomarkers in patients with T2DM receiving NS supplements and while some reductions in malondialdehyde and increases in total antioxidant capacity have been reported, the consistency and clinical significance of these findings are still uncertain. Thus, despite strong preclinical rationale, the extrapolation to human pathology requires cautious interpretation.

TQ is a powerful inhibitor of inflammatory processes [63]. In vitro and in vivo studies show that TQ reduces the production of nitrogen oxides (NO) and proinflammatory cytokines (TNF-α, IL-6, IL-1β) by inhibiting the nuclear factor NF-κB and the inhibition of the IRAK1 protein [64,65,66,67,68]. This inhibition thus significantly reduces the activity of inflammatory cells, contributing to the protection of tissues affected by diabetic complications. By inhibiting inflammation factors and protecting pancreatic tissues, a decrease in systemic inflammation is achieved that contributes to the relief of diabetic symptoms [65,69]. Still, in clinical contexts, the anti-inflammatory effects of NS remain largely inferential, as few studies have directly quantified inflammatory mediators such as TNF-α or IL-6 in diabetic patients. Some small-scale clinical trials have shown minor decreases in inflammatory cytokines, but the results were not always statistically significant. These inconsistencies suggest that the pronounced anti-inflammatory actions observed in preclinical models might not fully translate under human physiological conditions, possibly due to differences in pharmacokinetics or the complexity of systemic inflammation in T2DM.

**Figure 3:**
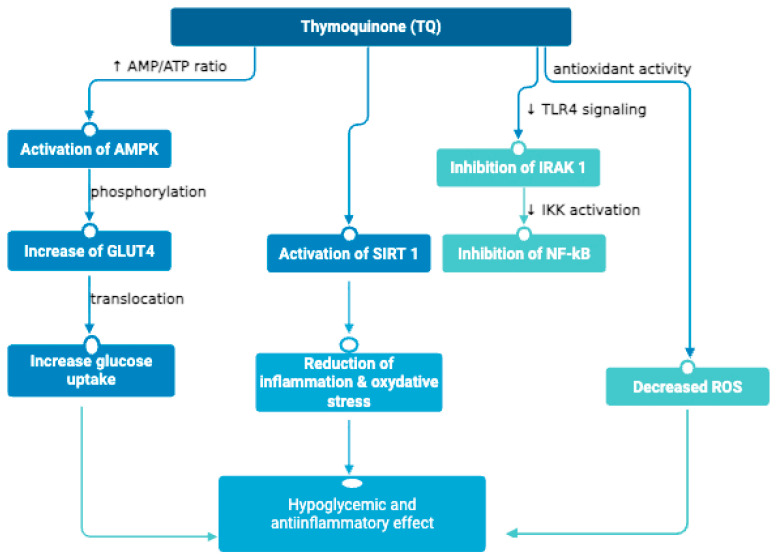
Flowchart of the main pharmacological mechanisms of TQ.

Figure 3 shows a flowchart that summarizes the main molecular mechanisms of TQ, highlighting the central role of TQ in activating signaling pathways that regulate energy metabolism and reduce inflammation, critical processes in ameliorating diabetic complications.

Thymoquinone is a monoterpene benzoquinone compound, first isolated from NS seeds in the 1960s [70], and is synthesized in plants from γ-terpinene in the secondary metabolism stage [71]. TQ accounts for 28 to 57% of the volatile oil, the exact composition being dependent on the species, the chemotype of the seeds, and the method of oil extraction [30,72].

TQ is a yellow crystalline substance, having a molar mass of 164.20 g/mol, and the molecular formula C_10_H_12_O_2_. TQ has been shown to have antioxidant, anti-inflammatory, analgesic, antimicrobial, antineoplastic, antihypertensive, hypoglycemic, and hepatoprotective properties [49,69,70,72,73,74,75,76,77]. The activity of TQ to neutralize oxidants has been attributed to the ease with which it crosses cell membranes to reach intracellular targets. Depending on the conditions of the cellular environment, TQ can undergo enzymatic or non-enzymatic redox reactions, leading to the production of antioxidants (thymohydroquinone) that have the role of neutralizing free radicals [75].

Thymohydroquinone (THQ) is the thymoquinol or 2,5-Dihydroxy-para-cymene. It has a molecular weight of 166.22 g/mol, and the toxicity of the compound is LD50 = 25 mg/kg [78,79]. THQ is the phenolic hydroquinone derivative of TQ, being the first reductive product of TQ. THQ exhibits vast and powerful intermolecular interactions, forming an infinite network of hydrogen bonds in its crystalline structure. Some studies have concluded that monoterpenes (thymol and carvacrol) are used to obtain THQ by cytochrome P-450 enzymes, which is later converted into TQ [80].

THQ has been analyzed and studied for its antibacterial properties against several bacteria: *Staphylococcus aureus, Shigella flexneri, Pseudomonas aeruginosa, Escherichia coli, Salmonella typhimurium,* and *Salmonella enteritidis* [81]. It has also been investigated by the DPPH method and the ORAC test in terms of outstanding antioxidant and anti-inflammatory activity [30,82]. Although NS and its bioactive constituents are generally regarded as safe, dose-dependent adverse effects have been reported in preclinical toxicity studies. At high doses, TQ has demonstrated hepatotoxic and nephrotoxic effects in rodents, indicating a narrow therapeutic window at supraphysiological concentrations. Therefore, the overall safety profile should be considered dose- and context-dependent rather than uniformly benign.

Dithymoquinone (DTQ) is a photodimer of TQ; it is also called nigellone, and has the molecular formula C_2_OH_24_O_4_, and the molecular weight 328.17 g/mol. DTQ was detected in lower concentrations in NS oil. Esharkawy and colab. conducted an in vitro study to evaluate the antioxidant, antifungal, and antitumor potential of DTQ compared to TQ. In the survey, DTQ was obtained by photo dimming reaction from TQ, as it is found in trace amounts in NS oil. Both compounds were evaluated for antioxidant, antifungal, and cytotoxic properties, and the results confirmed these properties; the pharmacological activity was superior for TQ [78]. Another in vitro study investigated the antiviral potential of DTQ, in which quantum chemical descriptors were correlated with experimental biological activity. The in vitro antiviral activity of DTQ was identified after screening, exhibiting both nuclear receptor ligand and enzyme inhibition activities [81].

Thymol (2-isopropyl-5-methylphenol) is a phenolic monoterpene characterized by its low solubility in water, hydrophobic properties, and non-selective cytotoxic effects, as well as anti-inflammatory, antifungal, and antibacterial activities [83]. A study conducted by Azizi et al. on rodent models highlights antioxidant and neuroprotective effects by improving oxidative damage and attenuating cognitive impairments due to thymol’s property to inhibit oxidative stress at the cellular level [84]. Another study conducted by Agarwal (2020) notes the antidiabetic and antioxidant activity following thymol supplementation in the diet of diabetic rats [85].

γ-terpinene is a monoterpenoid that exhibits strong antioxidant and anti-inflammatory activity, with involvement in modulating cytokine release and a significant role in reducing inflammation. It also has antinociceptive and analgesic properties [86]. It accumulates in immature seeds and is a precursor to p-cymen, being gradually replaced by carvacrol, THQ, and TQ as the seeds develop. These constituents are found almost exclusively in the shell of seeds; the composition of monoterpenes changes during seed development [87]. From γ-terpinene, carvacrol is biosynthesized in plants via mevalonate or by oxidation by cytochrome P450 monooxygenases. The formation of γ-terpinene is essential in the synthesis of thymol, which is an isomer of carvacrol [88,89,90].

P-cymene (4-isopropyltoluene) or p-cymol is a monoterpene, alkyl-substituted, woody-smelling, slightly pungent aromatic compound precursor to carvacrol, which is naturally found in essential oils and exhibits antioxidant activity by capturing reactive species (hydroxyl radical and nitric oxide) and antitumor, along with a variety of other pharmacological benefits, including antimicrobials against Gram-positive and Gram-negative bacteria [87].

Rodrigo de Oliveira Formiga et al. highlighted the role of the antioxidant mechanism and immunomodulation, the anti-inflammatory effect of p-cimene involving the cytoprotection of the intestinal barrier, the maintenance of the mucus layer of the intestine, and the preservation of communication junctions, thus leading to the improvement of intestinal inflammation [91,92]. Other studies have highlighted analgesic/antinociceptive and anti-inflammatory properties by blocking the signaling pathways of AMPK and NF-kB and inhibiting cytokine signal expression and production of cytokines, tumor necrosis factor α (TNF-α), interleukin 1β (IL-1β), interleukin 6 (IL-6), and interleukin 10 (IL-10) [87,93].

P-cymene has been found to attenuate acute induced lung injury by decreasing the infiltration of proinflammatory cytokines and inflammatory cells in murine study models. The anti-inflammatory capacity of p-cymene was demonstrated in another study in which it reduced the production of TNF-α and IL-1β in infected mice and murine cell lines [94].

Due to its therapeutic potential, p-cymene is utilized in biomedical applications, as it is one of the most effective monoterpenes with neuroprotective properties, characterized by its ability to decrease caspase-3 expression in neurons and exhibit a significant antineurodegenerative role [95,96]. The protective mechanism of p-cymene on the pathogenesis of atherosclerosis has also been demonstrated by its action within lipid metabolism, being considered a novel antiatherosclerotic agent [97].

Carvacrol (5-isopropyl-2-methylphenol) is a naturally occurring phenolic compound, a monoterpenoid alcohol often found in plants along with its isomer, thymol, which exerts anti-inflammatory and antioxidant activities, manifested by preventing the peroxidation of polyunsaturated fatty acids by inducing superoxide dismutase (SOD) and CAT, also by decreasing the number of proinflammatory cytokines. The broad spectrum of activity of carvacrol includes antidiabetic, analgesic, anti-obesity, cardioprotective, renoprotective, anticonvulsant and neuroprotective, anticancer, and modulation of the immune response, as well as hepatoprotective properties [88,98,99,100,101]. Table 1 summarizes the essential therapeutic properties of the bioactive constituents of NS.

The literature presents solid evidence on the efficacy of the *Nigella sativa* L. species, both in vitro studies (laboratory studies) and in vivo studies, carried out on animal models (experimental studies) and on human subjects (clinical studies).

### 2.1. In vitro Studies on the Antidiabetic Potential of NS

The antioxidant and antidiabetic capacity of NS seed extract was documented in an in vitro study conducted in 2022 by Veeramani et al., the antidiabetic activity being confirmed by enzymatic testing (α-amylase method), and the thymoquinone present in the seed extract was identified and characterized by Fourier transformation infrared spectrometry (FTIR) and confirmed by anthraquinone testing. The antioxidant ability was established by the 2,2-diphenylpicrylhydrazyl (DPPH) uptake activity test [56].

Another in vitro study conducted in 2021 by Dalli et al. investigates the protective properties of TQ against diabetes by inducing a reduction in oxidative stress. In the study, bioactive compounds were identified by HPLC-UV and GC-MS methods. The conclusions of the investigation revealed the inhibitory capacity of the NS extract on α-amylase (digestive enzyme) and on the glucose absorption activity in the intestine [58].

Following the in vitro study carried out in 2021 by Tiji et al., the hypoglycemic effect of NS extract was revealed through the inhibitory action of digestive enzymes, α-glucosidase and α-amylase, detected as effective therapeutic targets for balancing pathological postprandial blood glucose. Thus, two types of NS extracts with different polarities, based on hexane and acetone, respectively, were investigated by the GC/MS and HPLC/DAD methods (a spectroscopic technique used for quantitative determinations). The inhibition capacity of the acetone-based NS extract was superior to that of the hexane-based NS extract and comparable to that of the standard drug, acarbose. The resulting conclusions support the use of NS seed extracts for the prevention and management of DM by capitalizing on the inhibiting effect of carbohydrate enzymes, intestinal α-glucosidase, and pancreatic α-amylase [55].

Another relevant in vitro study by Vijayakumar et al. was performed in 2021 on the aqueous NS seed extract, in which techniques such as FTIR (to confirm the active compounds of the extract), high-resolution transmission electron microscopy (HR-TEM), and energy dispersive X-ray analysis (EDX) were used to confirm the purity of the compound. In addition to the significant antidiabetic activity recorded by inhibiting the enzymes that ensure the hydrolysis of carbohydrates (alpha-glucosidase, alpha-amylase, and dipeptidyl-peptidase IV), the antibacterial potential against Gram-negative bacteria, the free radical scavenging, and the anti-inflammatory property were determined by the cell membrane stabilization action [57].

### 2.2. In vivo Studies on the Antidiabetic Potential of NS

A notable study conducted by Ali (2021) examined the antihyperglycemic activity of TQ in combination with Metformin in humans (60 patients with T2DM in a 90-day randomized trial) and in diabetic mouse models (21 days) [158]. In this study, a reduction in blood glucose was found in mice given a combination of TQ and Metformin compared to those given Metformin alone. While the combined TQ–Metformin treatment showed improved outcomes in both animal and human models, the magnitude of glycemic reduction was considerably smaller in the clinical group compared with the preclinical models. This contrast underscores the common translational limitation of phytochemical research, where biological activity observed under controlled experimental conditions does not fully predict therapeutic efficacy in patients. Furthermore, most available human studies are short-term and use small sample sizes, making it difficult to draw definitive conclusions about long-term metabolic or microvascular benefits.

A marked decrease in HbA1c, fasting blood glucose, and postprandial blood glucose was observed in patients receiving standard therapy with Metformin [158]. Table 2 summarizes the main studies carried out in recent years on animal models used in biomedical research and the results of these investigations.

Table 3 summarizes some studies carried out on human subjects, allowing the evaluation of the efficacy and safety of the various ways of administering NS, essential in the deepening of research in T2DM therapeutics.

These findings of the studies suggest the substantial importance of NS (TQ), which induces a hypoglycemic and normalizing effect on glycemic indices. The data presented indicate that both preclinical and clinical studies support the hypothesis that NS can act effectively in reducing blood glucose levels and improving metabolic parameters. The summarization of the main results of studies regarding the antidiabetic effects of NS and its marker compound, TQ, is consistent with the data in the scientific literature on its use in alternative medicine and highlights its potential in supporting modern diabetic therapy [176]. Overall, although in vitro and animal studies provide strong mechanistic support for the antidiabetic properties of NS, clinical evidence remains preliminary and sometimes contradictory. More standardized, large-scale clinical trials are needed to confirm that the biochemical effects of thymoquinone observed preclinically can be effectively harnessed in human diabetes management.

## 3. *Nigella sativa* L.’s Evidence of T2DM Complications

Numerous studies have investigated the properties and biological effects of NS in the management of T2DM, demonstrating both the benefits on glycemic control and the protective activity in terms of diabetic complications. A topical product in the form of an NS-based ointment was investigated in the management of diabetic peripheral neuropathy by Khodaie (2024), which confirmed the relieving effects in neuropathic patients [177].

In an experimental study conducted by Alkhalaf (2020) [178] on 50 rats with glycosuria and hyperglycemia, the properties of NS extract to improve both hyperglycemia and neuroinflammation and oxidative stress, leading to apoptosis, were brought to attention. The study aimed to explore the beneficial effect of NS in diabetic neuropathy in terms of serum glucose levels, insulin, as well as inflammatory biomarkers and oxidative parameters. Hyperglycemia induces the autooxidation of glucose and, implicitly, the increase in ROS production. The histopathological findings were made by observing brain cells in rats with diabetic neuropathy [178].

Another study also conducted on rats by Alrafiah (2021) elucidated the paramount importance of TQ in regulating oxidative stress (by increasing the level of antioxidant enzymes) and the inflammatory process (by attenuating inflammatory cytokines) in the cerebellum, thus preventing neuronal damage [179]. The neuroprotective activity of TQ by combating oxidative neuroinflammation and neuroapoptosis in rats has also been investigated by Famurewa (2024) and Okoh (2025) in in vivo studies [180,181].

Other studies on the beneficial effect of TQ in neuronal injury were conducted on mice by Abo Mansour (2023) and Ateș (2025) [65,182].

TQ’s ability to accelerate the healing of lesions in retinal pigment epithelial cells, which may be caused by oxidative stress or arterial obstruction, was documented in a study conducted by Sen and Kasikci (2023) on retinal pigment epithelium cell lines [183].

The nephroprotective properties of TQ have also been investigated in diabetic rat studies by Mohebbati (2020), Nehar (2021), and Ayaz (2023), whose results obtained on kidney tissues support efficacy in relieving diabetic nephropathy [164,166,184].

In summary, preclinical studies strongly indicate that NS and its bioactive compounds, especially thymoquinone, may improve diabetes through antioxidant, anti-inflammatory, and insulin-sensitizing effects. However, these findings have not been consistently confirmed in clinical trials. Translating the positive results seen in cell and animal studies to reliable benefits in humans remains challenging. Variations in dosage, extraction methods, formulations, and patient characteristics make comparisons between studies difficult and may explain the inconsistent clinical outcomes.

## 4. Evidence of *Nigella sativa* L. in Commonly Associated T2DM Conditions

In addition to the directly hypoglycemic effects, NS is also distinguished by the benefits brought in the management of conditions frequently associated with T2DM, various comorbidities: hypertension, stroke, myocardial infarction or other cardiovascular diseases, dyslipidemia, liver disease, and obesity. Multiple studies have highlighted the beneficial effects of NS as an adjunctive therapy in hypertensive patients to reduce cardiovascular risks. One such randomized, double-blind, placebo-controlled clinical trial was conducted by Parisa Shoaei-Hagh (2021) [185], where systolic and diastolic blood pressure, fasting blood glucose, and blood lipid panel were assessed during the 8 weeks of NS oil treatment, twice daily. The value of systolic blood pressure was significantly reduced compared to baseline, thus indicating the promising therapeutic potential of NS as a complementary treatment for the control of blood glucose and lipid metabolism in hypertensive patients [185].

In addition to the effects on glucose metabolism, the administration of NS oil demonstrated the potential to improve the lipid profile and reduce blood pressure in patients with metabolic syndrome, significant risk factors in T2DM [186]. Thus, NS may contribute to reducing cardiovascular risk in chronic diabetic patients. The significant impact of NS and TQ in mitigating oxidative stress and inflammatory processes, as well as on apoptotic parameters after myocardial infarction induction, has been documented by Raluca Maria Pop (2024), Medhet (2022), Bocsan (2021), Rathod (2022), and Adiyaman (2022) in rat studies [187,188,189,190,191].

Hafez (2024) noted the property of TQ to relieve diabetes-induced liver damage and hyperlipidemia, in a rat study, by regulating oxidative-nitrosative stress, inflammation, and apoptosis [192]. Other studies evaluating the hepatoprotective effect of TQ, improving lipid metabolism, restoring anti-inflammatory markers and antioxidant enzymes, were conducted by Owumi (2025) on 40 rats and by Almatroodi (2021) on 32 diabetic rats [167,193].

The beneficial therapeutic potential of NS in obesity and non-alcoholic fatty liver disease has been explored by Bashir (2023) in an 80-mouse study, as well as by Esmail (2021) and Ramineedu (2024) in other studies [52,194,195]. The high-fat diet can cause obesity, and within it, the adipocytes secrete adipokines that cause chronic inflammation and various induced metabolic pathologies. The marked role of NS in the management of overweight and inflammation was reported in Razmpoosh’s (2024) study of 46 eligible participants that examined the effects of NS administration on IL-1β, IL-6, and leptin and on insulin parameters in obese women [196]. Some studies have noted the complexity of the relationship between obesity and neurodegenerative disorders [197,198,199], chronic inflammation, oxidative stress, and insulin resistance, exacerbating neuronal cell damage [200].

Obesity is correlated with an increased level of reactive oxygen species and reduced antioxidant defense mechanisms, which can trigger the evolution of inflammatory processes and thus constitute a risk factor for the initiation of cancer [12,201,202]. The exploration of the antitumor potential of TQ was carried out by Ravi (June 2025) in an in silico and cytotoxicity study on human cell lines, in which TQ exhibited a multi-target anticancer mechanism by inhibiting the key pathways involved in cancer pathology (proliferation, survival, and metastasis), reinforcing its promising therapeutic potential [203]. The anticancer activity of TQ is also supported by the study of Gnanasekaran (2021) and the study of Alsanosi (2022) [204,205], which explored the potential of NS as an epigenetic therapy for cancer, as an alternative to synthetic drugs, and which concluded that TQ constitutes a valuable therapeutic strategy for both solid and blood tumors. TQ inhibited cell proliferation and induced apoptosis in cancer cells, ensuring the regulation of tumor cell epigenome [204,205].

NS is of remarkable importance in the pathology of patients with T2DM involving cardiometabolic parameters such as: glycemic factors (fasting insulin, HbA1c, homeostatic model evaluation for insulin resistance HOMA-IR, homeostatic model evaluation for HOMA-β pancreatic beta cells), lipid panel (HDL, LDL, TG, TC), anthropometric indices (BMI, body weight), liver enzymes (AST, ALT) and creatinine, as documented by Karimi in a recent study (June 2025), concluding that NS supplementation improved glycemic markers and lipid profiles [206,207]. Ke Jiahan (2023) also identified various mechanisms underlying lipotoxicity and metabolic changes in the diabetic heart, requiring effective and precise cardioprotective therapy for diabetic cardiomyopathy [208].

## 5. Innovations in the Ways of NS Administration

The clinical administration of TQ has been limited due to its low oral bioavailability and hydrophobic nature [209]. Innovative approaches were explored, including various nanotechnological formulas such as nanoemulsions [210], nanostructured lipid carriers [211,212], liposomal formulations, and nanosystems [213,214,215]. The formulation of TQ in lipid nanoparticles gives it a superior bioavailability, the inclusion in delivery systems of the active substance ensuring a targeted distribution and improving its therapeutic efficacy [216].

Nanotechnology is considered a desirable solution for conventional drug therapy. It bridges the gap between biological and physical sciences, providing innovative drug delivery systems, protecting the active substance from metabolic degradation, and facilitating absorption through the intestine [217]. A notable technological advance and a remarkable advance in diabetes research is the development of nano sensors for monitoring blood glucose levels, ensuring better accuracy through increased sensitivity [218]. Computer-assisted nanomedicine and virtual screening offer a promising perspective in exploring structure-activity relationships and elucidating nano-bio interactions, guiding the rational design and directed evolution of nanomedicines [219].

Another therapeutic approach involved incorporating TQ into bacterial cellulose, utilizing cyclodextrin as a solubilizer for the hydrophobic compound, to create an effective dressing for treating wounds and managing infections in dermal wounds [220]. Innovative approaches have also been noted, such as a natural therapy to relieve diabetic foot ulcers with the integration of modern science, in the form of hydrogels or silver nanoparticles that have been targeted at the wound microbiome, immune response, and controlled release of the natural active substance [221].

An innovative formulation was presented by Xue and Lin (2024) [222] using a natural polysaccharide, chitosan, obtained by partial deacetylation of chitin. Starting from TQ and chitosan (a cationic polymer), through the electrostatic self-assembly process (which involves the attraction between molecules with opposite electrical charges), a supramolecular structure with biomedical applicability was created. TQ was incorporated into the lipid core of the nanoparticles, which were then coated with a layer of chitosan. The efficacy of the nano-sized product in managing pediatric diabetes cases was evaluated through in vitro antidiabetic and functional cell culture tests, which concluded that TQ-Chitosan nanoparticles can be safely administered to pediatric diabetic patients [222]. Self-assembled nanoparticles are an advanced technology in the field of nanomedicine and a formulation optimized from a physicochemical and therapeutic point of view, with promising prospects in raising the standards of modern treatments.

## 6. Safety and Adverse Effects of NS

Clinical evaluations and experimental studies have shown that supplementation with NS, especially in the form of oil or standardized extracts, has an excellent safety profile [223]. Studies have reported increased tolerability, with no significant adverse effects on hepatic, renal, or gastrointestinal functions, the level of toxicity being correlated with the dose administered, route of administration, and duration of administration [224,225]. Also, the history of traditional use gives more confidence in the safety of this herb used as a remedy in the complementary therapy of diabetes, TQ having a wide therapeutic window and being safer administered orally than administered intraperitoneally, aspects included in a study on rats of the toxicological profile of TQ, carried out by Mashayekhi-Sardo [42].

A study to evaluate the safety and efficacy of TQ was conducted by Dr. Ahmed Kaseb in the United States of America (California, Florida, and Texas), over 6 months, on 60 patients who received a daily dose of 3 g of NS oil. The study is available on the ClinicalTrials.gov platform and has been completed [226].

The evaluation of the safety and toxicological profile of TQ was conducted in Li’s (2023) study on mice [227], which were randomly divided into two groups and injected intraperitoneally. The study’s conclusions indicated low toxicity and significant efficacy [227].

The safety and therapeutic efficacy of TQ were also investigated by Allemailem (2021) in a mouse study [228], where the administration of liposomal TQ reduced the bacterial load in lung tissues in mice infected with multidrug-resistant *Acinetobacter baumannii*, demonstrating time- and dose-dependent activity, and TQ having broad-spectrum antimicrobial activity. The results showed higher efficacy due to liposomes and low toxicity of TQ when incorporated into liposomes [228].

Another notable study is the one conducted by Sanpinit (2023) [229], which evaluated the oral toxicity of NS oil after daily administration over a period of 28 days. The oral toxicological study was carried out on rats, which were randomly assigned to three experimental groups. The results of this in vivo study on subacute toxicity indicate that oral administration of NS oil is safe and without adverse effects in rats [229].

## 7. Future Directions of Research as a Supplement in T2DM

The current consistent evidence for the use of NS is promising, but further research is still needed to address: establishing the optimal dose (determining the maximum effective doses as well as the optimal mode of administration to maximize therapeutic benefits without compromising patient safety), studies in diverse patient groups (including underrepresented populations and assessing inter-individual variations in response to treatment), integration into conventional therapies (further research on the interactions between NS and standard antidiabetic drugs, to optimize therapeutic synergies), clarification of molecular mechanisms (investigations at the molecular level to identify precisely the pathways by which NS influences glucose homeostasis and pancreatic function).

Timely prospects include the development of well-defined treatment regimens and their effective integration into conventional therapies targeting issues related to doses and pharmacological interactions. This review highlights that NS is a valuable adjunctive and complementary therapy option for patients with T2DM, suggesting that further research could lead to the development of new, modern, and innovative therapeutic strategies.

## 8. Conclusions

Research shows that *Nigella sativa* L. can be successfully included in complementary and alternative therapy for T2DM pathology, having multiple benefits both in diseases associated with T2DM and in complications of T2DM. These findings highlight the potential of this species as a valuable source due to its various pharmacological properties. Although NS has long been celebrated in traditional medicine as a universal remedy, modern evidence supports only a subset of these claims. Rigorous clinical trials are essential to substantiate its promising, yet still preliminary, therapeutic potential suggested by preclinical data.

## Figures and Tables

**Table 1:** The essential therapeutic properties of the bioactive constituents of NS.

Bioactive Compounds	Therapeutic Properties	References
Thymoquinone	Anti-inflammatory Antioxidant Anti-nociceptive Hypoglycemic Hepatoprotective Neuroprotective Antitumoral Antimicrobial Immunomodulatory Antihistamine	[102,103,104,105,106,107,108,109,110,111,112,113,114,115,116,117,118,119,120,121,122,123,124,125,126,127]
Thymohidroquinone	Antibacterial Antioxidant Anti-inflammatory activity	[81,89,128,129]
Dithymoquinone	Antioxidant Antifungals Antitumoral Antiviral	[78,81,130]
Thymol	Anti-inflammatory Antifungal Antimicrobial activity Antioxidant Neuroprotective Antidiabetic	[90,131,132,133,134,135]
γ-terpinen	Antioxidant Anti-inflammatory Anti-nociceptive Analgesic	[86,136,137]
p-Cymene	Antioxidant Antitumoral Antimicrobial Anti-inflammatory Neuroprotective Anti-atherosclerotic	[132,133,138,139,140,141,142,143,144]
Carvacrol	Anti-inflammatory Antioxidant Antidiabetic Analgesic Cardioprotective Reno-protective Antimicrobial Neuroprotective	[131,145,146,147,148,149,150,151,152,153,154,155,156,157]

**Table 2:** Studies on laboratory animal models.

Author of the Study	Study	Dose Administered	Period	Observed Effects
Khan and Zaidi (2024) [159]	Rats	100 mg/kg body weight NS extract 10 mg/kg body weight thymoquinone	28 days	Significant decrease in blood glucose, total cholesterol and low-density lipoprotein; Protective effect of NS seed extract and TQ on diabetic rats
Fadishei (2021) [160]		2 mg/kg body weight TQ		
	Rats with metabolic disorder	Daily peritoneal injection	54 days	Decreased lipid profile, hepatic enzymes, insulin and the blood pressure
Akhtar (2020) [73]	Rabbits	2.5 mL/kg body weight NS seed oil	24 days	NS oil treatment lowered serum blood glucose levels and lipid contents, total cholesterol, triglycerides and low density lipoprotein cholesterol
		Oral administration		
Sadiq (2021) [161]		0.5 mL NS	Significant decrease in blood glucose and partial regeneration	
	Rats	1 mL NS 1.5 mL NS Oral administration	40 days of beta islet cells of the pancreas	
Faisal Lutfi (2021) [162]	Rats	50 mg/kg body weight TQ Daily	4 weeks	Decreased glycated hemoglobin (HbA1c) level, lipid peroxidase and nitric oxide (NO); higher TAC in diabetic rats treated with TQ and attenuated of diabetic nephropathy
Dong (2020) [163]	Mice	140 mg/kg 70 mg/kg 35 mg/kg, (0.1 mL/10 g) NS	4 weeks	NS reduced blood glucose levels, triglycerides, total cholesterol and LDL-C; antihyperglycemic and antihyperlipidemic action after the NS treatment
Mohebbati (2020) [164]	Rats	200 mg/kg NS extract 400 mg/kg NS extract Orally	6 weeks	Decreased serum glucose level and improved the lipid level in rats treated with NS; Increased antioxidant status (CAT) after NS administration
Mostafa (2025) [165]	Rats	40 mg/kg body weight TQ, orally via stomach tube	4 weeks	TQ balanced HbA1c levels and insulin resistance, controlled inflammatory cytokines IL-1β, IL-6, TNF-α and CRP
Ayaz (2023) [166]	Wistar Albino rats	2.5 mL/kg body weight NS oil	56 days	Amelioration of hyperglycemia and pathological renal changes caused by diabetes; NS can favorably regulate oxidative stress; NS proves nephroprotective effect and anti-apoptotic potential
Almatroodi (2021) [167]	Diabetic rats	150 mg/kg body weight TQ	8 weeks	Improvement of serum glucose levels and insulin, improving lipid metabolism (TC, TG, LDL-C);
		Orally		TQ confirms the essential role in the antidiabetic activity

Abbreviations: NS = *Nigella sativa*; TQ = Thymoquinone; TAC = total antioxidant capacity; LDL-C = low density lipoprotein-cholesterol; CAT = catalase; CRP = C-reactive protein; TC = total cholesterol; TG = triglycerides.

**Table 3:** Studies on human subjects.

Author of the Study	Subjects	Dose Administered	Period	Observed Effects
Rahmani (2022) [168]	46 diabetic patients	2 g/day NS oil	12 weeks	Notable improvement in serum HbA1c and fasting blood sugar levels (FBS); NS significantly increased antioxidant levels (SOD, TAC)
Kooshki (2020) [169]	50 patients	1000 mg NS oil as two capsules	8 weeks	Significant decrease in fasting blood glucose and improved lipid panel (TC, TG, LDL-C)
Hadi (2021) [170]	43 patients	500 mg NS oil twice daily	8 weeks	Favorable action on glycemic control body weight in diabetic patients and improvement of lipid parameters
El-Afify (2025) [171]	60 pediatric diabetic patients	450 mg NS oil, Twice daily	3 months	Notable decrease in cholesterol and LDL-C levels in pediatric patients with type 1 diabetes; Representative reduction in MDA and nitric oxide levels, highlighting the ability of NS to eliminate free radicals
Jangjo- Borazjani (2023) [17]	40 patients with type 2 diabetes	2 g NS capsules (crushed seeds)	8 weeks	Significant decrease in insulin level, LDL-C and C-reactive protein; Improvement of diabetic biomarkers
Rao (2020) [172]	40 subjects	4.7 g NS + 0.75 g fenugreek Twice daily	12 weeks	Pronounced decrease in postprandial blood glucose, FBG and HbA1c after twelve weeks of treatment
Mahmoudian (2025) [173]	103 adolescent girls	1000 mg NS/day	16 weeks	Improving the glycemic profile of adolescent girls (FBG, plasma glucose 2-h postprandial)
Ghods (2024) [14]	80 patients	2 g NS-Cuminum cyminum Soft gel	12 weeks	Pronounced decrease in FBG and HbA1c levels compared to placebo; NS-CC oil may decrease blood glucose levels and insulin resistance
Ammar (2021) [174]	127 prediabetic patients	500 mg TQ + 500 mg Metformin Three times daily	6 months	Significant weight loss, improving glycemic control, better body fat distribution (waist circumference, body mass index), achieving normal oxidative balance; Increased serum superoxide dismutase activity
Mostafa (2021) [175]	117 obese prediabetic subjects	450 mg 2x/day NS oil soft gelatin capsules	6 months	Favorable effects on glycemic parameters; Improving lipid panel
				Suppression of inflammation (TNF-α)

Abbreviations: NS = *Nigella sativa*; FBS = fasting blood sugar; SOD = superoxide dismutase; TAC = total antioxidant capacity; TC = total cholesterol; TG = triglycerides; LDL-C = low-density lipoprotein cholesterol; FBG = fasting blood glucose; NS-CC = combination of *Nigella sativa* and *Cuminum cyminum*; TQ = thymoquinone.

## Data Availability

The original contributions presented in the study are included in the article. Further inquiries can be directed to the corresponding author.

## Abbreviations

The following abbreviations are used in this manuscript:

T2DM	Type 2 diabetes mellitus
NS	*Nigella sativa*
TQ	Thymoquinone
THQ	Thymohydroquinone
DTQ	Dithymoquinone

## References

[1] Salmerón-Manzano E., Garrido-Cardenas J.A., Manzano-Agugliaro F. (2020). Worldwide Research Trends on Medicinal Plants. Int. J. Environ. Res. Public. Health.

[2] Tönnies T., Rathmann W., Hoyer A., Brinks R., Kuss O. (2021). Quantifying the Underestimation of Projected Global Diabetes Prevalence by the International Diabetes Federation (IDF) Diabetes Atlas. BMJ Open Diabetes Res. Care.

[3] Kundnani N.R., Lolescu B., Dinu A.-R., Berceanu-Vaduva D.M., Dumitrescu P., Tamaș T.-P., Sharma A., Popa M.-D. (2024). Biotechnology Revolution Shaping the Future of Diabetes Management. Biomolecules.

[4] Burlou-Nagy C., Bănică F., Jurca T., Vicaș L.G., Marian E., Muresan M.E., Bácskay I., Kiss R., Fehér P., Pallag A. (2022). *Echinacea purpurea* (L.) Moench: Biological and Pharmacological Properties. A Review. Plants.

[5] Ansari P., Akther S., Hannan J.M.A., Seidel V., Nujat N.J., Abdel-Wahab Y.H.A. (2022). Pharmacologically Active Phytomolecules Isolated from Traditional Antidiabetic Plants and Their Therapeutic Role for the Management of Diabetes Mellitus. Molecules.

[6] Duarte A.M., Guarino M.P., Barroso S., Gil M.M. (2020). Phytopharmacological Strategies in the Management of Type 2 Diabetes Mellitus. Foods.

[7] Tran N., Pham B., Le L. (2020). Bioactive Compounds in Anti-Diabetic Plants: From Herbal Medicine to Modern Drug Discovery. Biology.

[8] Rydén L., Lindsten J. (2021). The History of the Nobel Prize for the Discovery of Insulin. Diabetes Res. Clin. Pract..

[9] Lu Y., Wang W., Liu J., Xie M., Liu Q., Li S. (2023). Vascular Complications of Diabetes: A Narrative Review. Medicine.

[10] Balbaa M., El-Zeftawy M., Abdulmalek S.A. (2021). Therapeutic Screening of Herbal Remedies for the Management of Diabetes. Molecules.

[11] Behl T., Gupta A., Albratty M., Najmi A., Meraya A.M., Alhazmi H.A., Anwer M.K., Bhatia S., Bungau S.G. (2022). Alkaloidal Phytoconstituents for Diabetes Management: Exploring the Unrevealed Potential. Molecules.

[12] Adam S.H., Abu I.F., Kamal D.A.M., Febriza A., Kashim M.I.A.M., Mokhtar M.H. (2023). A Review of the Potential Health Benefits of *Nigella sativa* on Obesity and Its Associated Complications. Plants.

[13] Derosa G., D’Angelo A., Maffioli P., Cucinella L., Nappi R.E. (2024). The Use of *Nigella sativa* in Cardiometabolic Diseases. Biomedicines.

[14] Ghods M., Karimi S., Salari S., Alem E., Bahmani P., Karimi A., Saeedpour A., Noormohamadi M., Jahromi S.R. (2024). Anti-diabetic Effect of a Combination of Black Seed (*Nigella sativa*) and Cumin (*Cuminum cyminum*), a Two-step Study from Bench to Bed. Funct. Food Sci. Online.

[15] Singh T.G., Sharma R., Kaur A., Dhiman S., Singh R. (2020). Evaluation of renoprotective potential of Ficus religiosa in attenuation of diabetic nephropathy in rats. Obes. Med..

[16] Pishdad R., Auwaerter P.G., Kalyani R.R. (2024). Diabetes, SGLT-2 Inhibitors, and Urinary Tract Infection: A Review. Curr. Diab. Rep..

[17] Jangjo-Borazjani S., Dastgheib M., Kiyamarsi E., Jamshidi R., Rahmati-Ahmadabad S., Helalizadeh M., Iraji R., Cornish S.M., Mohammadi-Darestani S., Khojasteh Z. (2023). Effects of resistance training and *Nigella sativa* on type 2 diabetes: Implications for metabolic markers, low-grade inflammation and liver enzyme production. Arch. Physiol. Biochem..

[18] Adhikari B. (2021). Roles of Alkaloids from Medicinal Plants in the Management of Diabetes Mellitus. J. Chem..

[19] Maurya A., Mohan S., Verma S.C. (2021). Antidiabetic Potential of Naturally Occurring Sesquiterpenes: A Review. Curr. Top. Med. Chem..

[20] Feng J., Wang X., Ye X., Ares I., Lopez-Torres B., Martínez M., Martínez-Larrañaga M.-R., Wang X., Anadón A., Martínez M.-A. (2022). Mitochondria as an important target of metformin: The mechanism of action, toxic and side effects, and new therapeutic applications. Pharmacol. Res..

[21] Zhou Y., Xie Y., Dong J., He K., Che H. (2024). Insulin Resistance-Nutritional Index: A Simple Index and Potential Predictor of Mortality Risk in Patients with Chronic Heart Failure and Type 2 Diabetes. Diabetes Metab. Syndr. Obes..

[22] Alam S., Sarker M.M.R., Sultana T.N., Chowdhury M.N.R., Rashid M.A., Chaity N.I., Zhao C., Xiao J., Hafez E.E., Khan S.A. (2022). Antidiabetic Phytochemicals From Medicinal Plants: Prospective Candidates for New Drug Discovery and Development. Front. Endocrinol..

[23] Khalivulla S.I., Mohammed A., Mallikarjuna K. (2021). Novel Phytochemical Constituents and their Potential to Manage Diabetes. Curr. Pharm. Des..

[24] Kumar S., Mittal A., Babu D., Mittal A. (2021). Herbal Medicines for Diabetes Management and its Secondary Complications. Curr. Diabetes Rev..

[25] Simon-Szabó L., Lizák B., Sturm G., Somogyi A., Takács I., Németh Z. (2024). Molecular Aspects in the Development of Type 2 Diabetes and Possible Preventive and Complementary Therapies. Int. J. Mol. Sci..

[26] Salehi B., Ata A., V. Anil Kumar N., Sharopov F., Ramírez-Alarcón K., Ruiz-Ortega A., Abdulmajid Ayatollahi S., Valere Tsouh Fokou P., Kobarfard F., Amiruddin Zakaria Z. (2019). Antidiabetic Potential of Medicinal Plants and Their Active Components. Biomolecules.

[27] Rasouli H., Yarani R., Pociot F., Popović-Djordjević J. (2020). Anti-diabetic potential of plant alkaloids: Revisiting current findings and future perspectives. Pharmacol. Res..

[28] Ahmad M.F., Ahmad F.A., Ashraf S.A., Saad H.H., Wahab S., Khan M.I., Ali M., Mohan S., Hakeem K.R., Athar M.T. (2021). An updated knowledge of Black seed (*Nigella sativa* Linn.): Review of phytochemical constituents and pharmacological properties. J. Herb. Med..

[29] Aslani M.R., Saadat S., Boskabady M.H. (2024). Comprehensive and updated review on anti-oxidant effects of *Nigella sativa* and its constituent, thymoquinone, in various disorders. Iran. J. Basic Med. Sci..

[30] Salehi B., Quispe C., Imran M., Ul-Haq I., Živković J., Abu-Reidah I.M., Sen S., Taheri Y., Acharya K., Azadi H. (2021). Nigella Plants–Traditional Uses, Bioactive Phytoconstituents, Preclinical and Clinical Studies. Front. Pharmacol..

[31] Ojueromi O.O., Oboh G., Ademosun A.O. (2022). Black Seed (*Nigella sativa*): A Favourable Alternative Therapy for Inflammatory and Immune System Disorders. Inflammopharmacology.

[32] Adam S.H., Mohd Nasri N., Kashim M.I.A.M., Abd Latib E.H., Ahmad Juhari M.A.A., Mokhtar M.H. (2022). Potential health benefits of *Nigella sativa* on diabetes mellitus and its complications: A review from laboratory studies to clinical trials. Front. Nutr..

[33] Alberts A., Moldoveanu E.-T., Niculescu A.-G., Grumezescu A.M. (2024). *Nigella sativa*: A Comprehensive Review of Its Therapeutic Potential, Pharmacological Properties, and Clinical Applications. Int. J. Mol. Sci..

[34] Sam J.H., Chan Y.S., Siner A. (2024). Antioxidant and Antimalarial Properties of *Nigella sativa*. Int. J. Chem. Eng. Appl..

[35] Koshak D.A.E. (2021). Effects of *Nigella sativa* as a Treatment of Patients With Upper Respiratory Tract Infection Caused by SARS-coronavirus-2: A Prospective, Randomized, Open-label, Controlled Clinical Study. Clin. Gov..

[36] Nyulas K.-I., Simon-Szabó Z., Pál S., Fodor M.-A., Dénes L., Cseh M.J., Barabás-Hajdu E., Csipor B., Szakács J., Preg Z. (2024). Cardiovascular Effects of Herbal Products and Their Interaction with Antihypertensive Drugs—Comprehensive Review. Int. J. Mol. Sci..

[37] Hamed E., Toaima W., Abd El-Aleem W. (2023). Impact of Different Planting Locations on *Nigella sativa* L. Yield in Egypt. Egypt. J. Desert Res..

[38] Ahmad R., Ahmad N., Amir M., AlJHISI F., Alamer M.H., Al-Shaban H.R., Alsultan B.M., Alsadah Z.A., Aldawood N.A., Chathoth S. (2020). Variation in *Nigella sativa* quality and its standardization via instrumental analysis: A study based on geographical origin. Not. Bot. Horti Agrobot. Cluj-Napoca.

[39] Belgaumi U.I., Patil S., Gandhi J.M., Shete A.S. (2020). The Many Therapeutic Applications of *Nigella sativa*–A Review of Literature. J. Evol. Med. Dent. Sci..

[40] Ferizi R., Ramadan M.F., Maxhuni Q. (2023). Black Seeds (*Nigella sativa*) Medical Application and Pharmaceutical Perspectives. J. Pharm. Bioallied Sci..

[41] Page M.J., McKenzie J.E., Bossuyt P.M., Boutron I., Hoffmann T.C., Mulrow C.D., Shamseer L., Tetzlaff J.M., Moher D. (2021). Updating guidance for reporting systematic reviews: Development of the PRISMA 2020 statement. J. Clin. Epidemiol..

[42] Mashayekhi-Sardoo H., Rezaee R., Karimi G. (2020). An overview of in vivo toxicological profile of thymoquinone. Toxin Rev..

[43] Mohebbati R., Abbasnezhad A. (2020). Effects of *Nigella sativa* on endothelial dysfunction in diabetes mellitus: A review. J. Ethnopharmacol..

[44] Elhariri S., Burud I., Zulaimy N.A., Tong J.A., Ahmed I., Kar Chun S.C., Kumaran P. (2024). Systematic Review of Randomized Controlled Trials in Uses of *Nigella sativa* (Black Seed) in Metabolic Syndrome. W. Afr. J. Med..

[45] Ahmad A., Alqahtani S., Jan B.L., Raish M., Rabba A.K., Alkharfy K.M. (2020). Gender effect on the pharmacokinetics of thymoquinone: Preclinical investigation and in silico modeling in male and female rats. Saudi Pharm. J..

[46] Karimi Z., Mirza Alizadeh A., Ezzati Nazhad Dolatabadi J., Dehghan P. (2019). *Nigella sativa* and its Derivatives as Food Toxicity Protectant Agents. Adv. Pharm. Bull..

[47] Kavyani Z., Musazadeh V., Golpour-hamedani S., Moridpour A.H., Vajdi M., Askari G. (2023). The effect of *Nigella sativa* (black seed) on biomarkers of inflammation and oxidative stress: An updated systematic review and meta-analysis of randomized controlled trials. Inflammopharmacology.

[48] Koshak A.E., Koshak E.A., Mobeireek A.F., Badawi M.A., Wali S.O., Malibary H.M., Atwah A.F., Alhamdan M.M., Almalki R.A., Madani T.A. (2021). *Nigella sativa* for the treatment of COVID-19: An open-label randomized controlled clinical trial. Complement. Ther. Med..

[49] Shaukat A., Zaidi A., Anwar H., Kizilbash N. (2023). Mechanism of the antidiabetic action of *Nigella sativa* and Thymoquinone: A review. Front. Nutr..

[50] Mahomoodally M.F., Aumeeruddy M.Z., Legoabe L.J., Montesano D., Zengin G. (2022). *Nigella sativa* L. and Its Active Compound Thymoquinone in the Clinical Management of Diabetes: A Systematic Review. Int. J. Mol. Sci..

[51] Ibrahim K.G., Hudu S.A., Jega A.Y., Taha A., Yusuf A.P., Usman D., Adeshina K.A., Umar Z.U., Nyakudya T.T., Erlwanger K.H. (2024). Thymoquinone: A comprehensive review of its potential role as a monotherapy for metabolic syndrome. Iran. J. Basic. Med. Sci..

[52] Bashir K.M.I., Kim J.W., Kim J.-K., Chun Y.-S., Choi J.-S., Ku S.-K. (2023). Efficacy Confirmation Test of Black Cumin (*Nigella sativa* L.) Seeds Extract Using a High-Fat Diet Mouse Model. Metabolites.

[53] Maideen N.M.P. (2021). Antidiabetic Activity of *Nigella sativa* (Black Seeds) and Its Active Constituent (Thymoquinone): A Review of Human and Experimental Animal Studies. Chonnam Med. J..

[54] Alqahtani A.S., Hidayathulla S., Rehman M.T., ElGamal A.A., Al-Massarani S., Razmovski-Naumovski V., Alqahtani M.S., El Dib R.A., AlAjmi M.F. (2019). Alpha-Amylase and Alpha-Glucosidase Enzyme Inhibition and Antioxidant Potential of 3-Oxolupenal and Katononic Acid Isolated from *Nuxia oppositifolia*. Biomolecules.

[55] Tiji S., Bouhrim M., Addi M., Drouet S., Lorenzo J.M., Hano C., Bnouham M., Mimouni M. (2021). Linking the Phytochemicals and the α-Glucosidase and α-Amylase Enzyme Inhibitory Effects of *Nigella sativa* Seed Extracts. Foods.

[56] Veeramani S., Narayanan A.P., Yuvaraj K., Sivaramakrishnan R., Pugazhendhi A., Rishivarathan I., Jose S.P., Ilangovan R. (2022). *Nigella sativa* flavonoids surface coated gold NPs (Au-NPs) enhancing antioxidant and anti-diabetic activity. Process Biochem..

[57] Vijayakumar S., Divya M., Vaseeharan B., Chen J., Biruntha M., Silva L.P., Durán-Lara E.F., Shreema K., Ranjan S., Dasgupta N. (2021). Biological Compound Capping of Silver Nanoparticle with the Seed Extracts of Blackcumin (*Nigella sativa*): A Potential Antibacterial, Antidiabetic, Anti-inflammatory, and Antioxidant. J. Inorg. Organomet. Polym. Mater..

[58] Dalli M., Daoudi N.E., Azizi S.-E., Benouda H., Bnouham M., Gseyra N. (2021). Chemical Composition Analysis Using HPLC-UV/GC-MS and Inhibitory Activity of Different *Nigella sativa* Fractions on Pancreatic α-Amylase and Intestinal Glucose Absorption. BioMed Res. Int..

[59] Varghese L.N., Mehrotra N. (2020). α-Amylase inhibitory activity of microencapsulated *Nigella sativa* L. and herb- drug interaction: An in vitro analysis. Ann. Phytomed. Int. J..

[60] Alkis H., Demir E., Taysi M.R., Sagir S., Taysi S. (2021). Effects of *Nigella sativa* oil and thymoquinone on radiation-induced oxidative stress in kidney tissue of rats. Biomed. Pharmacother..

[61] Ali M.Y., Akter Z., Mei Z., Zheng M., Tania M., Khan M.A. (2021). Thymoquinone in autoimmune diseases: Therapeutic potential and molecular mechanisms. Biomed. Pharmacother..

[62] Mahmud N.M., Paraoan L., Khaliddin N., Kamalden T.A. (2022). Thymoquinone in Ocular Neurodegeneration: Modulation of Pathological Mechanisms via Multiple Pathways. Front. Cell. Neurosci..

[63] Liu Y., Huang L., Kim M.-Y., Cho J.Y. (2022). The Role of Thymoquinone in Inflammatory Response in Chronic Diseases. Int. J. Mol. Sci..

[64] Ahmad A., Alkharfy K.M., Jan B.L., Ahad A., Ansari M.A., Al-Jenoobi F.I., Raish M. (2020). Thymoquinone treatment modulates the Nrf2/HO-1 signaling pathway and abrogates the inflammatory response in an animal model of lung fibrosis. Exp. Lung Res..

[65] Ateş Ş., Ülger H., Uçar S., Okan A., Ocak M., Güvenilir E., Şükranlı Z.Y., Kaymak E., Doğanyiğit Z., Taheri S. (2025). Evaluation of the Effects of Thymoquinone on RAGE/NOX4 Expressions and Brain Tissue Morphometry in Experimental Alzheimer’s Disease Induced by Amyloid Beta 1-42 Peptide. Biomolecules.

[66] Hossen M.J., Yang W.S., Kim D., Aravinthan A., Kim J.-H., Cho J.Y. (2017). Thymoquinone: An IRAK1 inhibitor with in vivo and in vitro anti-inflammatory activities. Sci. Rep..

[67] Salah A., Sleem R., Abd-Elaziz A., Khalil H. (2023). Regulation of NF-κB Expression by Thymoquinone; A Role in Regulating Pro-Inflammatory Cytokines and Programmed Cell Death in Hepatic Cancer Cells. Asian Pac. J. Cancer Prev. APJCP.

[68] Talebi M., Talebi M., Farkhondeh T., Samarghandian S. (2021). Biological and therapeutic activities of thymoquinone: Focus on the Nrf2 signaling pathway. Phytother. Res..

[69] Chatterjee G., Saha A.K., Khurshid S., Saha A. (2025). A Comprehensive Review of the Antioxidant, Antimicrobial, and Therapeutic Efficacies of Black Cumin (*Nigella sativa* L.) Seed Oil and Its Thymoquinone. J. Med. Food.

[70] Malik S., Singh A., Negi P., Kapoor V.K. (2021). Thymoquinone: A small molecule from nature with high therapeutic potential. Drug Discov. Today.

[71] Ahmad A., Mishra R.K., Vyawahare A., Kumar A., Rehman M.U., Qamar W., Khan A.Q., Khan R. (2019). Thymoquinone (2-Isopropyl-5-methyl-1, 4-benzoquinone) as a chemopreventive/anticancer agent: Chemistry and biological effects. Saudi Pharm. J..

[72] Rahman M.T. (2020). Potential benefits of combination of *Nigella sativa* and Zn supplements to treat COVID-19. J. Herb. Med..

[73] Akhtar M.T., Qadir R., Bukhari I., Ashraf R.A., Malik Z., Zahoor S., Murtaza M.A., Siddique F., Shah S.N.H., Saadia M. (2020). Antidiabetic potential of *Nigella sativa* L seed oil in alloxaninduced diabetic rabbits. Trop. J. Pharm. Res..

[74] Khodaie S.-A., Razavi R., Nikkhah H., Namiranian N., Kamalinejad M. (2024). *Nigella sativa* L. and its bioactive and nutraceutical components in the management of diabetic peripheral neuropathy. Inflammopharmacology.

[75] Gomathinayagam R., Ha J.H., Jayaraman M., Song Y.S., Isidoro C., Dhanasekaran D.N. (2020). Chemopreventive and Anticancer Effects of Thymoquinone: Cellular and Molecular Targets. J. Cancer Prev..

[76] Akter Z., Ahmed F.R., Tania M., Khan M.A. (2021). Targeting Inflammatory Mediators: An Anticancer Mechanism of Thymoquinone Action. Curr. Med. Chem..

[77] Pop R.M., Sabin O., Suciu Ș., Vesa S.C., Socaci S.A., Chedea V.S., Bocsan I.C., Buzoianu A.D. (2020). *Nigella sativa*’s Anti-Inflammatory and Antioxidative Effects in Experimental Inflammation. Antioxidants.

[78] Elsharkawy E.R., Abdallah E.M., Markb A.A. (2021). Potential Cytotoxic, Antifungal, and Antioxidant Activity of Dithymoquinone and Thymoquinone. J. Hunan Univ. Nat. Sci..

[79] PubChem. https://pubchem.ncbi.nlm.nih.gov/.

[80] Sakib R., Caruso F., Aktar S., Belli S., Kaur S., Hernandez M., Rossi M. (2023). Antioxidant Properties of Thymoquinone, Thymohydroquinone and Black Cumin (*Nigella sativa* L.) Seed Oil: Scavenging of Superoxide Radical Studied Using Cyclic Voltammetry, DFT and Single Crystal X-ray Diffraction. Antioxidants.

[81] Esharkawy E.R., Almalki F., Hadda T.B. (2022). In vitro potential antiviral SARS-CoV-19- activity of natural product thymohydroquinone and dithymoquinone from *Nigella sativa*. Bioorganic Chem..

[82] Tesarova H., Svobodova B., Kokoska L., Marsik P., Pribylova M., Landa P., Vadlejch J. (2011). Determination of oxygen radical absorbance capacity of black cumin (*Nigella sativa*) seed quinone compounds. Nat. Prod. Commun..

[83] Sturabotti E., Moldoveanu V.G., Camilli A., Martinelli A., Simonetti G., Valletta A., Serangeli I., Giustini A., Miranda E., Migneco L.M. (2023). Thymol-Functionalized Hyaluronic Acid as Promising Preservative Biomaterial for the Inhibition of *Candida albicans* Biofilm Formation. ACS Macro Lett..

[84] Azizi Z., Salimi M., Amanzadeh A., Majelssi N., Naghdi N. (2020). Carvacrol and Thymol Attenuate Cytotoxicity Induced by Amyloid β25-35 Via Activating Protein Kinase C and Inhibiting Oxidative Stress in PC12 Cells. Iran. Biomed. J..

[85] Agarwal S., Tripathi R., Mohammed A., Rizvi S.I., Mishra N. (2020). Effects of thymol supplementation against type 2 diabetes in streptozotocin-induced rat model. Plant Arch..

[86] Bagińska S., Golonko A., Świsłocka R., Lewandowski W. (2024). Monoterpenes as Medicinal Agents: Exploring the Pharmaceutical Potential of p-Cymene, p-Cymenene, and γ-Terpinene. Acta Pol. Pharm..

[87] Balahbib A., El Omari N., Hachlafi N.E., Lakhdar F., El Menyiy N., Salhi N., Mrabti H.N., Bakrim S., Zengin G., Bouyahya A. (2021). Health beneficial and pharmacological properties of p-cymene. Food Chem. Toxicol..

[88] Mączka W., Twardawska M., Grabarczyk M., Wińska K. (2023). Carvacrol—A Natural Phenolic Compound with Antimicrobial Properties. Antibiotics.

[89] Krause S.T., Liao P., Crocoll C., Boachon B., Förster C., Leidecker F., Wiese N., Zhao D., Wood J.C., Buell C.R. (2021). The biosynthesis of thymol, carvacrol, and thymohydroquinone in Lamiaceae proceeds via cytochrome P450s and a short-chain dehydrogenase. Proc. Natl. Acad. Sci. USA.

[90] Tohidi B., Rahimmalek M., Arzani A., Trindade H. (2020). Sequencing and variation of terpene synthase gene (*TPS2*) as the major gene in biosynthesis of thymol in different Thymus species. Phytochemistry.

[91] Jin H., Leng Q., Zhang C., Zhu Y., Wang J. (2021). P-cymene prevent high-fat diet-associated colorectal cancer by improving the structure of intestinal flora. J. Cancer.

[92] Formiga R.d.O., Alves Júnior E.B., Vasconcelos R.C., Guerra G.C.B., Antunes de Araújo A., Carvalho T.G.D., Garcia V.B., de Araújo Junior R.F., Gadelha F.A.A.F., Vieira G.C. (2020). p-Cymene and Rosmarinic Acid Ameliorate TNBS-Induced Intestinal Inflammation Upkeeping ZO-1 and MUC-2: Role of Antioxidant System and Immunomodulation. Int. J. Mol. Sci..

[93] Pyo Y., Jung Y.J. (2024). Microbial Fermentation and Therapeutic Potential of p-Cymene: Insights into Biosynthesis and Antimicrobial Bioactivity. Fermentation.

[94] Ciesielska-Figlon K., Daca A., Kokotkiewicz A., Łuczkiewicz M., Zabiegała B., Witkowski J.M., Lisowska K.A. (2022). The influence of *Nigella sativa* essential oil on proliferation, activation, and apoptosis of human T lymphocytes in vitro. Biomed. Pharmacother..

[95] Olufunmilayo E.O., Gerke-Duncan M.B., Holsinger R.M.D. (2023). Oxidative Stress and Antioxidants in Neurodegenerative Disorders. Antioxidants.

[96] Wojtunik-Kulesza K., Rudkowska M., Kasprzak-Drozd K., Oniszczuk A., Borowicz-Reutt K. (2021). Activity of Selected Group of Monoterpenes in Alzheimer’s Disease Symptoms in Experimental Model Studies-A Non-Systematic Review. Int. J. Mol. Sci..

[97] Yang J., Zhong C., Yu J. (2023). Natural Monoterpenes as Potential Therapeutic Agents against Atherosclerosis. Int. J. Mol. Sci..

[98] Neopane D., Kushwaha P. (2025). Carvacrol in asthma management: A comprehensive review of its therapeutic potential and mechanisms of action. Pharmacol. Rep..

[99] Marconi G.D., Della Rocca Y., Fonticoli L., Guarnieri S., Carradori S., Rajan T.S., Pizzicannella J., Diomede F. (2022). The Beneficial Effect of Carvacrol in HL-1 Cardiomyocytes Treated with LPS-G: Anti-Inflammatory Pathway Investigations. Antioxidants.

[100] Hoca M., Becer E., Vatansever H.S. (2024). Carvacrol is potential molecule for diabetes treatment. Arch. Physiol. Biochem..

[101] Cicalău G.I.P., Babes P.A., Calniceanu H., Popa A., Ciavoi G., Iova G.M., Ganea M., Scrobotă I. (2021). Anti-Inflammatory and Antioxidant Properties of Carvacrol and Magnolol, in Periodontal Disease and Diabetes Mellitus. Molecules.

[102] Dong J., Zhang X., Wang S., Xu C., Gao M., Liu S., Li X., Cheng N., Han Y., Wang X. (2021). Thymoquinone Prevents Dopaminergic Neurodegeneration by Attenuating Oxidative Stress Via the Nrf2/ARE Pathway. Front. Pharmacol..

[103] Isaev N.K., Chetverikov N.S., Stelmashook E.V., Genrikhs E.E., Khaspekov L.G., Illarioshkin S.N. (2020). Thymoquinone as a Potential Neuroprotector in Acute and Chronic Forms of Cerebral Pathology. Biochem. Biokhimiia.

[104] Kalam M.A., Raish M., Ahmed A., Alkharfy K.M., Mohsin K., Alshamsan A., Al-Jenoobi F.I., Al-Mohizea A.M., Shakeel F. (2017). Oral bioavailability enhancement and hepatoprotective effects of thymoquinone by self-nanoemulsifying drug delivery system. Mater. Sci. Eng. C Mater. Biol. Appl..

[105] Khan M.A., Younus H. (2018). Thymoquinone Shows the Diverse Therapeutic Actions by Modulating Multiple Cell Signaling Pathways: Single Drug for Multiple Targets. Curr. Pharm. Biotechnol..

[106] Isaev N.K., Genrikhs E.E., Stelmashook E.V. (2023). Antioxidant Thymoquinone and Its Potential in the Treatment of Neurological Diseases. Antioxidants.

[107] Modarresi Chahardehi A., Ojaghi H.R., Motedayyen H., Arefnezhad R. (2024). Nano-based formulations of thymoquinone are new approaches for psoriasis treatment: A literature review. Front. Immunol..

[108] Majdalawieh A.F., Fayyad M.W., Nasrallah G.K. (2017). Anti-cancer properties and mechanisms of action of thymoquinone, the major active ingredient of *Nigella sativa*. Crit. Rev. Food Sci. Nutr..

[109] Negi P., Rathore C., Sharma G., Singh B., Katare O.P. (2018). Thymoquinone a Potential Therapeutic Molecule from the Plant *Nigella sativa*: Role of Colloidal Carriers in its Effective Delivery. Recent. Pat. Drug Deliv. Formul..

[110] Nassar W.M., El-Kholy W.M., El-Sawi M.R., El-Shafai N.M., Alotaibi B.S., Ghamry H.I., Shukry M. (2023). Ameliorative Effect of Thymoquinone and Thymoquinone Nanoparticles against Diazinon-Induced Hepatic Injury in Rats: A Possible Protection Mechanism. Toxics.

[111] Sadeghi E., Imenshahidi M., Hosseinzadeh H. (2023). Molecular mechanisms and signaling pathways of black cumin (*Nigella sativa*) and its active constituent, thymoquinone: A review. Mol. Biol. Rep..

[112] Samad N., Manzoor N., Muneer Z., Bhatti S.A., Imran I. (2021). Reserpine-induced altered neuro-behavioral, biochemical and histopathological assessments prevent by enhanced antioxidant defence system of thymoquinone in mice. Metab. Brain Dis..

[113] Samarghandian S., Farkhondeh T., Samini F. (2018). A Review on Possible Therapeutic Effect of *Nigella sativa* and Thymoquinone in Neurodegenerative Diseases. CNS Neurol. Disord. Drug Targets.

[114] Sarkar C., Jamaddar S., Islam T., Mondal M., Islam M.T., Mubarak M.S. (2021). Therapeutic perspectives of the black cumin component thymoquinone: A review. Food Funct..

[115] Imam A.L., Okesina A.A., Sulaimon F.A., Imam A., Ibiyeye R.Y., Oyewole L.A., Biliaminu S.A., Shehu M., Alli A.O., Omoola O.O. (2024). Thymoquinone ameliorate oxidative stress, GABAergic neuronal depletion and memory impairment through Nrf2/ARE signaling pathway in the dentate gyrus following cypermethrin administration. BMC Neurosci..

[116] Kantar D., Acun A.D., Danışman B. (2022). Effects of thymoquinone on scopolamine-induced spatial and echoic memory changes through regulation of lipid peroxidation and cholinergic impairment. Behav. Brain Res..

[117] Tiwari G., Gupta M., Devhare L.D., Tiwari R. (2024). Therapeutic and Phytochemical Properties of Thymoquinone Derived from *Nigella sativa*. Curr. Drug Res. Rev..

[118] Tabeshpour J., Mehri S., Abnous K., Hosseinzadeh H. (2019). Neuroprotective Effects of Thymoquinone in Acrylamide-Induced Peripheral Nervous System Toxicity Through MAPKinase and Apoptosis Pathways in Rat. Neurochem. Res..

[119] Zahra N., Fatima S., Nazir A., Farrukh S.Y., Anwer A., Sarwar A., Aziz T., Al Asmari F., Nahari A.M., Jalal R.S. (2025). In vivo and in silico analysis of anti inflammatory, antipyretic and analgesic activity of methanolic extract of *Nigella sativa*. J. Mol. Histol..

[120] Glamočlija U., Padhye S., Špirtović-Halilović S., Osmanović A., Veljović E., Roca S., Novaković I., Mandić B., Turel I., Kljun J. (2018). Synthesis, Biological Evaluation and Docking Studies of Benzoxazoles Derived from Thymoquinone. Molecules.

[121] Fatima Shad K., Soubra W., Cordato D.J. (2021). The role of thymoquinone, a major constituent of *Nigella sativa*, in the treatment of inflammatory and infectious diseases. Clin. Exp. Pharmacol. Physiol..

[122] Eissa N., Alwattar J.K., Jayaprakash P., Chkier D., Ahmed A.O., Ahmed A., Rizwan R., Mujeeb S., Rahal M., Sadek B. (2025). The Effects of Novel Thymoquinone-Loaded Nanovesicles as a Promising Avenue to Modulate Autism Associated Dysregulation by Restoring Oxidative Stress in Autism in Mice. Int. J. Nanomedicine.

[123] Dahmash E.Z., Ali D.K., Alyami H.S., AbdulKarim H., Alyami M.H., Aodah A.H. (2022). Novel Thymoquinone Nanoparticles Using Poly(ester amide) Based on L-Arginine-Targeting Pulmonary Drug Delivery. Polymers.

[124] Cobourne-Duval M.K., Taka E., Mendonca P., Soliman K.F.A. (2018). Thymoquinone increases the expression of neuroprotective proteins while decreasing the expression of pro-inflammatory cytokines and the gene expression NFκB pathway signaling targets in LPS/IFNγ -activated BV-2 microglia cells. J. Neuroimmunol..

[125] Ceylan T., Akin A.T., Karabulut D., Tan F.C., Taşkiran M., Yakan B. (2023). Therapeutic effect of thymoquinone on brain damage caused by nonylphenol exposure in rats. J. Biochem. Mol. Toxicol..

[126] Bin Abdulrahman K., Bamosa A., Bukhari A., Siddiqui I., Arafa M., Mohsin A., Althageel M., Aljuaeed M., Aldeailej I., Alrajeh A. (2022). The Effect of Short Treatment with *Nigella sativa* on Symptoms, the Cluster of Differentiation (CD) Profile, and Inflammatory Markers in Mild COVID-19 Patients: A Randomized, Double-Blind Controlled Trial. Int. J. Environ. Res. Public Health.

[127] Badary O.A., Hamza M.S., Tikamdas R. (2021). Thymoquinone: A Promising Natural Compound with Potential Benefits for COVID-19 Prevention and Cure. Drug Des. Devel. Ther..

[128] Shoaib A., Javed S., Wahab S., Azmi L., Tabish M., Sultan M.H., Abdelsalam K., Alqahtani S.S., Ahmad M.F. (2023). Cellular, Molecular, Pharmacological, and Nano-Formulation Aspects of Thymoquinone-A Potent Natural Antiviral Agent. Molecules.

[129] Wahab S., Alsayari A. (2023). Potential Pharmacological Applications of Nigella Seeds with a Focus on *Nigella sativa* and Its Constituents against Chronic Inflammatory Diseases: Progress and Future Opportunities. Plants.

[130] Tiwari P., Jena S., Satpathy S., Sahu P.K. (2019). *Nigella sativa*: Phytochemistry, Pharmacology and its Therapeutic Potential. Res. J. Pharm. Technol..

[131] Rathod N.B., Kulawik P., Ozogul F., Regenstein J.M., Ozogul Y. (2021). Biological activity of plant-based carvacrol and thymol and their impact on human health and food quality. Trends Food Sci. Technol..

[132] Hossain M.S., Sharfaraz A., Dutta A., Ahsan A., Masud M.A., Ahmed I.A., Goh B.H., Urbi Z., Sarker M.M.R., Ming L.C. (2021). A review of ethnobotany, phytochemistry, antimicrobial pharmacology and toxicology of *Nigella sativa* L.. Biomed. Pharmacother. Biomedecine Pharmacother..

[133] Abbas M., Gururani M.A., Ali A., Bajwa S., Hassan R., Batool S.W., Imam M., Wei D. (2024). Antimicrobial Properties and Therapeutic Potential of Bioactive Compounds in *Nigella sativa*: A Review. Molecules.

[134] Ouattar H., Zouirech O., Kara M., Assouguem A., Almutairi S.M., Al-Hemaid F.M., Rasheed R.A., Ullah R., Abbasi A.M., Aouane M. (2022). In Vitro Study of the Phytochemical Composition and Antioxidant, Immunostimulant, and Hemolytic Activities of *Nigella sativa* (Ranunculaceae) and Lepidium sativum Seeds. Molecules.

[135] Sampaio L.A., Pina L.T.S., Serafini M.R., Tavares D.D.S., Guimarães A.G. (2021). Antitumor Effects of Carvacrol and Thymol: A Systematic Review. Front. Pharmacol..

[136] Gheorghita D., Robu A., Antoniac A., Antoniac I., Ditu L.M., Raiciu A.-D., Tomescu J., Grosu E., Saceleanu A. (2022). In Vitro Antibacterial Activity of Some Plant Essential Oils against Four Different Microbial Strains. Appl. Sci..

[137] Sousa L.G.V., Castro J., Cavaleiro C., Salgueiro L., Tomás M., Palmeira-Oliveira R., Martinez-Oliveira J., Cerca N. (2022). Synergistic effects of carvacrol, α-terpinene, γ-terpinene, ρ-cymene and linalool against Gardnerella species. Sci. Rep..

[138] Shafodino F.S., Lusilao J.M., Mwapagha L.M. (2022). Phytochemical characterization and antimicrobial activity of *Nigella sativa* seeds. PLoS ONE.

[139] Caputo L., Amato G., De Martino L., De Feo V., Nazzaro F. (2023). Anti-Cholinesterase and Anti-α-Amylase Activities and Neuroprotective Effects of Carvacrol and p-Cymene and Their Effects on Hydrogen Peroxide Induced Stress in SH-SY5Y Cells. Int. J. Mol. Sci..

[140] Santos W.B.R., Melo M.A.O., Alves R.S., De Brito R.G., Rabelo T.K., Prado L.D.S., Silva V.K.d.S., Bezerra D.P., De Menezes-Filho J.E.R., Souza D.S. (2019). p-Cymene attenuates cancer pain via inhibitory pathways and modulation of calcium currents. Phytomedicine.

[141] Satira A., Espro C., Paone E., Calabrò P.S., Pagliaro M., Ciriminna R., Mauriello F. (2021). The Limonene Biorefinery: From Extractive Technologies to Its Catalytic Upgrading into p-Cymene. Catalysts.

[142] Seifi-Nahavandi B., Yaghmaei P., Ahmadian S., Ghobeh M., Ebrahim-Habibi A. (2020). Cymene consumption and physical activity effect in Alzheimer’s disease model: An in vivo and in vitro study. J. Diabetes Metab. Disord..

[143] Shareef S.H., Al-Medhtiy M.H., Ibrahim I.A.A., Alzahrani A.R., Jabbar A.A., Galali Y., Agha N.F.S., Aziz P.Y., Thabit M.A., Agha D.N.F. (2022). Gastroprophylactic Effects of p-Cymene in Ethanol-Induced Gastric Ulcer in Rats. Processes.

[144] Wang S., Wang X., Wang Y., Leng Q., Sun Y., Hoffman R.M., Jin H. (2021). The Anti-oxidant Monoterpene *p*-Cymene Reduced the Occurrence of Colorectal Cancer in a Hyperlipidemia Rat Model by Reducing Oxidative Stress and Expression of Inflammatory Cytokines. Anticancer Res..

[145] Khan I., Bahuguna A., Shukla S., Aziz F., Chauhan A.K., Ansari M.B., Bajpai V.K., Huh Y.S., Kang S.C. (2020). Antimicrobial potential of the food-grade additive carvacrol against uropathogenic E. coli based on membrane depolarization, reactive oxygen species generation, and molecular docking analysis. Microb. Pathog..

[146] Anaeigoudari A. (2022). Hepato- and reno-protective effects of thymoquinone, crocin, and carvacrol: A comprehensive review. Asian Pac. J. Trop. Biomed..

[147] Imran M., Aslam M., Alsagaby S.A., Saeed F., Ahmad I., Afzaal M., Arshad M.U., Abdelgawad M.A., El-Ghorab A.H., Khames A. (2022). Therapeutic application of carvacrol: A comprehensive review. Food Sci. Nutr..

[148] Hajiaghaalizadeh M., Sheikharabi M., Jazi M.S., Alhashem R., Hosseini S.S. (2025). Anti-biofilm activity of carvacrol-thymoquinone nanocarriers on vulvovaginal candidiasis isolates. Diagn. Microbiol. Infect. Dis..

[149] Addo K.A., Li H., Yu Y., Xiao X. (2023). Unraveling the mechanism of the synergistic antimicrobial effect of cineole and carvacrol on Escherichia coli O157:H7 inhibition and its application on fresh-cut cucumbers. Food Control.

[150] Azizi Z., Majlessi N., Choopani S., Naghdi N. (2022). Neuroprotective effects of carvacrol against Alzheimer’s disease and other neurodegenerative diseases: A review. Avicenna J. Phytomedicine.

[151] Asadi S., Nayeri-Fasaei B., Zahraei-Salehi T., Yahya-Rayat R., Shams N., Sharifi A. (2023). Antibacterial and anti-biofilm properties of carvacrol alone and in combination with cefixime against Escherichia coli. BMC Microbiol..

[152] Ghorani V., Alavinezhad A., Rajabi O., Mohammadpour A.H., Boskabady M.H. (2021). Safety and tolerability of carvacrol in healthy subjects: A phase I clinical study. Drug Chem. Toxicol..

[153] Khazdair M.R., Ghorani V., Boskabady M.H. (2022). Experimental and clinical evidence on the effect of carvacrol on respiratory, allergic, and immunologic disorders: A comprehensive review. BioFactors.

[154] Qu C., Li Z., Wang X. (2021). UHPLC-HRMS-Based Untargeted Lipidomics Reveal Mechanism of Antifungal Activity of Carvacrol against Aspergillus flavus. Foods.

[155] Souza G.H.d.A.d., Radai J.A.d.S., Vaz M.S.M., Silva K.E.d., Fraga T.L., Barbosa L.S., Simionatto S. (2021). In vitro and in vivo antibacterial activity assays of carvacrol: A candidate for development of innovative treatments against KPC-producing Klebsiella pneumoniae. PLoS ONE.

[156] Khazdair M.R., Moshtagh M., Anaeigoudari A., Jafari S., Kazemi T. (2024). Protective effects of carvacrol on lipid profiles, oxidative stress, hypertension, and cardiac dysfunction–A comprehensive review. Food Sci. Nutr..

[157] Wang J., Qin T., Chen K., Pan L., Xie J., Xi B. (2022). Antimicrobial and Antivirulence Activities of Carvacrol against Pathogenic Aeromonas hydrophila. Microorganisms.

[158] Ali S.M., Chen P., Sheikh S., Ahmad A., Ahmad M., Paithankar M., Desai B., Patel P., Khan M., Chaturvedi A. (2021). Thymoquinone with Metformin Decreases Fasting, Post Prandial Glucose, and HbA1c in Type 2 Diabetic Patients. Drug Res..

[159] Khan S.S., Zaidi K.U. (2024). Protective Effect of *Nigella sativa* Seed Extract and its BioactiveCompound Thymoquinone on Streptozotocin-induced Diabetic Rats. Cardiovasc. Hematol. Agents Med. Chem..

[160] Fadishei M., Ghasemzadeh Rahbardar M., Imenshahidi M., Mohajeri A., Razavi B.M., Hosseinzadeh H. (2021). Effects of *Nigella sativa* oil and thymoquinone against bisphenol A-induced metabolic disorder in rats. Phytother. Res..

[161] Sadiq N., Subhani G., Fatima S.A., Nadeem M., Zafer S., Mohsin M. (2021). Antidiabetic effect of *Nigella sativa* compared with metformin on blood glucose levels in streptozotocin induced diabetic albino wistar rats. Int. J. Basic Clin. Pharmacol..

[162] Faisal Lutfi M., Abdel-Moneim A.-M.H., Alsharidah A.S., Mobark M.A., Abdellatif A.A.H., Saleem I.Y., Al Rugaie O., Mohany K.M., Alsharidah M. (2021). Thymoquinone Lowers Blood Glucose and Reduces Oxidative Stress in a Rat Model of Diabetes. Molecules.

[163] Dong J., Liang Q., Niu Y., Jiang S., Zhou L., Wang J., Ma C., Kang W. (2020). Effects of *Nigella sativa* seed polysaccharides on type 2 diabetic mice and gut microbiota. Int. J. Biol. Macromol..

[164] Mohebbati R., Abbasnezhad A., Havakhah S., Mousavi M. (2020). The Effect of *Nigella sativa* on Renal Oxidative Injury in Diabetic Rats. Saudi J. Kidney Dis. Transplant..

[165] Mostafa M.D., Amer M.E., ElKomy M.A., Othman A.I., El--Missiry M.A. (2025). Thymoquinone controlled obesity and invigorated cognitive and memory performance in rats consuming a high-fat diet via modulating oxidative stress, inflammation and apoptosis. Sci. Rep..

[166] Ayaz H., Kaya S., Seker U., Nergiz Y. (2023). Comparison of the anti-diabetic and nephroprotective activities of vitamin E, metformin, and *Nigella sativa* oil on kidney in experimental diabetic rats. Iran. J. Basic Med. Sci..

[167] Almatroodi S.A., Alnuqaydan A.M., Alsahli M.A., Khan A.A., Rahmani A.H. (2021). Thymoquinone, the Most Prominent Constituent of *Nigella sativa*, Attenuates Liver Damage in Streptozotocin-Induced Diabetic Rats via Regulation of Oxidative Stress, Inflammation and Cyclooxygenase-2 Protein Expression. Appl. Sci..

[168] Rahmani A., Niknafs B., Naseri M., Nouri M., Tarighat-Esfanjani A. (2022). Effect of *Nigella sativa* Oil on Oxidative Stress, Inflammatory, and Glycemic Control Indices in Diabetic Hemodialysis Patients: A Randomized Double-Blind, Controlled Trial. Evid. Based Complement. Alternat. Med..

[169] Kooshki A., Tofighiyan T., Rastgoo N., Rakhshani M.H., Miri M. (2020). Effect of *Nigella sativa* oil supplement on risk factors for cardiovascular diseases in patients with type 2 diabetes mellitus. Phytother. Res..

[170] Hadi S., Daryabeygi-Khotbehsara R., Mirmiran P., McVicar J., Hadi V., Soleimani D., Askari G. (2021). Effect of *Nigella sativa* oil extract on cardiometabolic risk factors in type 2 diabetes: A randomized, double-blind, placebo-controlled clinical trial. Phytother. Res..

[171] El-Afify D., El Amrousy D. (2025). Cardioprotective Effect of *Nigella sativa* in Pediatric Patients with Type 1 Diabetes Mellitus: A Randomized Controlled Study. Pediatr. Drugs.

[172] Rao A.S., Hegde S., Pacioretty L.M., DeBenedetto J., Babish J.G. (2020). *Nigella sativa* and *Trigonella foenum-graecum* Supplemented Chapatis Safely Improve HbA1c, Body Weight, Waist Circumference, Blood Lipids, and Fatty Liver in Overweight and Diabetic Subjects: A Twelve-Week Safety and Efficacy Study. J. Med. Food.

[173] Mahmoudian A., Ashouri A., Mohammadzadeh F., Rahmani Bilandi R., Dashti S., Bahri N. (2025). Effect of *Nigella sativa*-L supplementation on glycemia in adolescent polycystic ovarian syndrome: Secondary analysis of a randomized controlled trial study. J. Ovarian Res..

[174] Ammar I.M.M., Salem M.A.A. (2021). Amelioration of polycystic ovary syndrome-related disorders by supplementation of thymoquinone and metformin. Middle East. Fertil. Soc. J..

[175] Mostafa T.M., Hegazy S.K., Elnaidany S.S., Shehabeldin W.A., Sawan E.S. (2021). *Nigella sativa* as a promising intervention for metabolic and inflammatory disorders in obese prediabetic subjects: A comparative study of *Nigella sativa* versus both lifestyle modification and metformin. J. Diabetes Complicat..

[176] Syuhada S., Anggadiredja K., Kurniati N.F., Akrom A. (2023). The Potential of *Nigella sativa* oil on Clinical output improvement of diabetic neuropathy. J. Appl. Pharm. Sci..

[177] Khodaie S.-A., Nikkhah H., Namiranian N., Abotorabi M., Askari M., Khalilzadeh S.H., Khatibi Aghda A., Kamalinejad M. (2024). Topical *Nigella sativa* L. product: A new candidate for the management of diabetic peripheral neuropathy. Inflammopharmacology.

[178] Alkhalaf M.I., Hussein R.H., Hamza A. (2020). Sinteza verde a nanoparticulelor de argint cu extract *de Nigella sativa* atenuează neuropatia diabetică prin efecte antiinflamatorii și antioxidante. Saudi J. Biol. Sci..

[179] Alrafiah A. (2021). Thymoquinone Protects Neurons in the Cerebellum of Rats through Mitigating Oxidative Stress and Inflammation Following High-Fat Diet Supplementation. Biomolecules.

[180] Famurewa A.C., Elsawy H., Sedky A. (2024). Thymoquinone Abrogates Acrylamide-Induced Cerebellar Toxicity via Modulation of Nuclear Factor Erythroid 2-Related Factor 2/Nuclear Factor Kappa B Signaling, Oxidative Neuroinflammation, and Neuroapoptosis in Rats. J. Med. Food.

[181] Okoh O.S., Akintunde J.K., Akamo A.J., Akpan U. (2025). Thymoquinone inhibits Neuroinflammatory mediators and vasoconstriction injury via NF-κB dependent NeuN/GFAP/Ki-67 in hypertensive Dams and F1 male pups on exposure to a mixture of Bisphenol-A analogues. Toxicol. Appl. Pharmacol..

[182] Abo Mansour H.E., Elberri A.I., Ghoneim M.E.-S., Samman W.A., Alhaddad A.A., Abdallah M.S., El-Berri E.I., Salem M.A., Mosalam E.M. (2023). The Potential Neuroprotective Effect of Thymoquinone on Scopolamine-Induced In Vivo Alzheimer’s Disease-like Condition: Mechanistic Insights. Molecules.

[183] Sen S., Kasikci M. (2023). Low-dose rosmarinic acid and thymoquinone accelerate wound healing in retinal pigment epithelial cells. Int. Ophthalmol..

[184] Nehar S., Rani P., Kumar C. (2021). Evaluation of genoprotective and antioxidative potentiality of ethanolic extract of N. sativa seed in streptozotocin induced diabetic albino rats. Vegetos.

[185] Shoaei-Hagh P., Kamelan Kafi F., Najafi S., Zamanzadeh M., Heidari Bakavoli A., Ramezani J., Soltanian S., Asili J., Hosseinzadeh H., Eslami S. (2021). A randomized, double-blind, placebo-controlled, clinical trial to evaluate the benefits of *Nigella sativa* seeds oil in reducing cardiovascular risks in hypertensive patients. Phytother. Res..

[186] Shirazi M., Khodakarami F., Feizabad E., Ghaemi M. (2020). The effects of *Nigella sativa* on anthropometric and biochemical indices in postmenopausal women with metabolic syndrome. Endocrine.

[187] Pop R.M., Vassilopoulou E., Jianu M.-E., Roșian Ș.H., Taulescu M., Negru M., Bercian C., Boarescu P.-M., Bocsan I.C., Feketea G. (2024). *Nigella sativa* oil attenuates inflammation and oxidative stress in experimental myocardial infarction. BMC Complement Med. Ther..

[188] Medhet M., El-Bakly W.M., Badr A.M., Awad A., El-Demerdash E. (2022). Thymoquinone attenuates isoproterenol-induced myocardial infarction by inhibiting cytochrome C and matrix metalloproteinase-9 expression. Clin. Exp. Pharmacol. Physiol..

[189] Bocsan I.C., Pop R.M., Sabin O., Sarkandy E., Boarescu P.-M., Roşian Ş.H., Leru P.M., Chedea V.S., Socaci S.A., Buzoianu A.D. (2021). Comparative Protective Effect of *Nigella sativa* Oil and Vitis vinifera Seed Oil in an Experimental Model of Isoproterenol-Induced Acute Myocardial Ischemia in Rats. Molecules.

[190] Rathod S., Agrawal Y., Sherikar A., Nakhate K.T., Patil C.R., Nagoor Meeran M.F., Ojha S., Goyal S.N. (2022). Thymoquinone Produces Cardioprotective Effect in β-Receptor Stimulated Myocardial Infarcted Rats via Subsiding Oxidative Stress and Inflammation. Nutrients.

[191] Adıyaman M.Ş., Adıyaman Ö.A., Dağlı A.F., Karahan M.Z., Dağlı M.N. (2022). Prevention of doxorubicin-induced experimental cardiotoxicity by *Nigella sativa* in rats. Rev. Port. Cardiol..

[192] Hafez M.H., Ez Elarab S.M., Tohamy H.G., El-Far A.H. (2024). Thymoquinone attenuates diabetes-induced hepatic damage in rat via regulation of oxidative/nitrosative stress, apoptosis, and inflammatory cascade with molecular docking approach. Sci. Rep..

[193] Owumi S., Otunla M., Akindipe P., Oluwawibe B., Babalola J.O., Chimezie J., Arunsi U., Owoeye O., Oyelere A.K. (2025). Thymoquinone modulates oxidative stress and inflammation, correcting mercury-induced HO-1/NRF/Trx pathway disruption in experimental rat hepatorenal system: An in vivo and in silico study. Biometals Int. J. Role Met. Ions Biol. Biochem. Med..

[194] Esmail M., Anwar S., Kandeil M., El-Zanaty A.M., Abdel-Gabbar M. (2021). Effect of *Nigella sativa*, atorvastatin, or L-Carnitine on high fat diet-induced obesity in adult male Albino rats. Biomed. Pharmacother..

[195] Ramineedu K., Sankaran K.R., Mallepogu V., Rendedula D.P., Gunturu R., Gandham S., Md S.I., Meriga B. (2024). Thymoquinone mitigates obesity and diabetic parameters through regulation of major adipokines, key lipid metabolizing enzymes and AMPK/p-AMPK in diet-induced obese rats. 3 Biotech.

[196] Razmpoosh E., Safi S., Mazaheri M., Khalesi S., Nazari M., Mirmiran P., Nadjarzadeh A. (2024). A crossover randomized controlled trial examining the effects of black seed (*Nigella sativa*) supplementation on IL-1β, IL-6 and leptin, and insulin parameters in overweight and obese women. BMC Complement. Med. Ther..

[197] Bittencourt A., Brum P.O., Ribeiro C.T., Gasparotto J., Bortolin R.C., De Vargas A.R., Heimfarth L., De Almeida R.F., Moreira J.C.F., De Oliveira J. (2022). High fat diet-induced obesity causes a reduction in brain tyrosine hydroxylase levels and non-motor features in rats through metabolic dysfunction, neuroinflammation and oxidative stress. Nutr. Neurosci..

[198] Kao Y.-C., Wei W.-Y., Tsai K.-J., Wang L.-C. (2019). High Fat Diet Suppresses Peroxisome Proliferator-Activated Receptors and Reduces Dopaminergic Neurons in the Substantia Nigra. Int. J. Mol. Sci..

[199] Schmitt L.O., Gaspar J.M. (2023). Obesity-Induced Brain Neuroinflammatory and Mitochondrial Changes. Metabolites.

[200] Neto A., Fernandes A., Barateiro A. (2023). The complex relationship between obesity and neurodegenerative diseases: An updated review. Front. Cell. Neurosci..

[201] Harborg S., Kjærgaard K.A., Thomsen R.W., Borgquist S., Cronin-Fenton D., Hjorth C.F. (2024). New Horizons: Epidemiology of Obesity, Diabetes Mellitus, and Cancer Prognosis. J. Clin. Endocrinol. Metab..

[202] Behrooz A.B., Cordani M., Fiore A., Donadelli M., Gordon J.W., Klionsky D.J., Ghavami S. (2024). The obesity-autophagy-cancer axis: Mechanistic insights and therapeutic perspectives. Semin. Cancer Biol..

[203] Ravi Y., Vethamoni P.I., Saxena S.N., Kaviyapriya M., Santhanakrishnan V.P., Raveendran M., Ashoka N.N., Choudhary S., Verma A.K., Harisha C.B. (2025). Anticancer potential of Thymoquinone from *Nigella sativa* L.: An in-silico and cytotoxicity study. PLoS ONE.

[204] Gnanasekaran P., Roy A., Sirpu Natesh N., Raman V., Ganapathy P., Arumugam M.K. (2021). Removal of microbial pathogens and anticancer activity of synthesized nano-thymoquinone from *Nigella sativa* seeds. Environ. Technol. Innov..

[205] Alsanosi S., Sheikh R.A., Sonbul S., Altayb H.N., Batubara A.S., Hosawi S., Al-Sakkaf K., Abdullah O., Omran Z., Alhosin M. (2022). The Potential Role of *Nigella sativa* Seed Oil as Epigenetic Therapy of Cancer. Molecules.

[206] Karimi M., Pirzad S., Pourfaraji S.M.A., Sedgi F.M., Darouei B., Amani-Beni R., Kazemi K., Rabiee R. (2025). Effects of black seed (*Nigella sativa* L.) on cardiometabolic indices in type 2 diabetic patients: A systematic review and meta-analysis of RCTs. Complement. Ther. Med..

[207] Saadati S., Naseri K., Asbaghi O., Abhari K., Zhang P., Li H.-B., Gan R.-Y. (2022). *Nigella sativa* supplementation improves cardiometabolic indicators in population with prediabetes and type 2 diabetes mellitus: A systematic review and meta-analysis of randomized controlled trials. Front. Nutr..

[208] Ke J., Pan J., Lin H., Gu J. (2023). Diabetic cardiomyopathy: A brief summary on lipid toxicity. ESC Heart Fail..

[209] Tekbaş A., Bremer-Streck S., Wissenbach D.K., Peters F.T., von Lilienfeld-Toal M., Soonawalla Z., Rauchfuß F., Settmacher U., Dahmen U. (2023). Gas Chromatography–Mass Spectrometry Detection of Thymoquinone in Oil and Serum for Clinical Pharmacokinetic Studies. Int. J. Mol. Sci..

[210] Shehata T.M., Almostafa M.M., Elsewedy H.S. (2022). Development and Optimization of *Nigella sativa* Nanoemulsion Loaded with Pioglitazone for Hypoglycemic Effect. Polymers.

[211] Zakarial Ansar F.H., Latifah S.Y., Wan Kamal W.H.B., Khong K.C., Ng Y., Foong J.N., Gopalsamy B., Ng W.K., How C.W., Ong Y.S. (2020). Pharmacokinetics and Biodistribution of Thymoquinone-loaded Nanostructured Lipid Carrier After Oral and Intravenous Administration into Rats. Int. J. Nanomedicine.

[212] Behzadifar S., Barras A., Plaisance V., Pawlowski V., Szunerits S., Abderrahmani A., Boukherroub R. (2023). Polymer-Based Nanostructures for Pancreatic Beta-Cell Imaging and Non-Invasive Treatment of Diabetes. Pharmaceutics.

[213] Rathore C., Rathbone M.J., Chellappan D.K., Tambuwala M.M., Pinto T.D.J.A., Dureja H., Hemrajani C., Gupta G., Dua K., Negi P. (2020). Nanocarriers: More than tour de force for thymoquinone. Expert. Opin. Drug Deliv..

[214] Shariare M.H., Khan M.A., Al-Masum A., Khan J.H., Uddin J., Kazi M. (2022). Development of Stable Liposomal Drug Delivery System of Thymoquinone and Its In Vitro Anticancer Studies Using Breast Cancer and Cervical Cancer Cell Lines. Molecules.

[215] Rathore C., Hemrajani C., Sharma A.K., Gupta P.K., Jha N.K., Aljabali A.A.A., Gupta G., Singh S.K., Yang J.-C., Dwivedi R.P. (2023). Self-nanoemulsifying drug delivery system (SNEDDS) mediated improved oral bioavailability of thymoquinone: Optimization, characterization, pharmacokinetic, and hepatotoxicity studies. Drug Deliv. Transl. Res..

[216] Allemailem K.S., Almatroudi A., Alrumaihi F., Aljaghwani A., Alnuqaydan A.M., Khalilullah H., Younus H., El-Kady A.M., Aldakheel F.M., Khan A.A. (2021). Antimicrobial, Immunomodulatory and Anti-Inflammatory Potential of Liposomal Thymoquinone: Implications in the Treatment of Bacterial Pneumonia in Immunocompromised Mice. Biomedicines.

[217] Vallianou N.G., Dalamaga M., Pavlou A., Rebelos E., Karamanolis N.N., Papachristoforou E., Mavrothalassitis E., Eleftheriadou I., Tentolouris N., Kounatidis D. (2025). The Transformative Role of Nanotechnology in the Management of Diabetes Mellitus: Insights from Current Research. Biomolecules.

[218] Manral K., Singh A., Singh Y. (2024). Nanotechnology as a potential treatment for diabetes and its complications: A review. Diabetes Metab. Syndr. Clin. Res. Rev..

[219] Shan X., Cai Y., Zhu B., Zhou L., Sun X., Xu X., Yin Q., Wang D., Li Y. (2024). Rational strategies for improving the efficiency of design and discovery of nanomedicines. Nat. Commun..

[220] Swingler S., Gupta A., Gibson H., Kowalczuk M., Adamus G., Heaselgrave W., Radecka I. (2022). Thymoquinone: Hydroxypropyl-β-cyclodextrin Loaded Bacterial Cellulose for the Management of Wounds. Pharmaceutics.

[221] Parveen R., Ali F., Singh S.D. (2024). Innovative Nanocomposites for Drug Delivery: A Novel Approach for Diabetic Foot Ulcer. Curr. Drug Deliv..

[222] Xue Q., Lin Y. (2024). In vitro and functional investigation reveals the curative effect of thymoquinone from black cumin-loaded chitosan nanoparticles on streptozotocin induced paediatric diabetes. Regen. Ther..

[223] Rahim M.A., Shoukat A., Khalid W., Ejaz A., Itrat N., Majeed I., Koraqi H., Imran M., Nisa M.U., Nazir A. (2022). A Narrative Review on Various Oil Extraction Methods, Encapsulation Processes, Fatty Acid Profiles, Oxidative Stability, and Medicinal Properties of Black Seed (*Nigella sativa*). Foods.

[224] Hannan M.A., Zahan M.S., Sarker P.P., Moni A., Ha H., Uddin M.J. (2021). Protective Effects of Black Cumin (*Nigella sativa*) and Its Bioactive Constituent, Thymoquinone against Kidney Injury: An Aspect on Pharmacological Insights. Int. J. Mol. Sci..

[225] Hannan M.A., Rahman M.A., Sohag A.A.M., Uddin M.J., Dash R., Sikder M.H., Rahman M.S., Timalsina B., Munni Y.A., Sarker P.P. (2021). Black Cumin (*Nigella sativa* L.): A Comprehensive Review on Phytochemistry, Health Benefits, Molecular Pharmacology, and Safety. Nutrients.

[226] (2024). Novatek Pharmaceuticals A Randomized, Double-Blind, Placebo-Controlled Study. To Evaluate the Safety and Efficacy of TQ Formula in Treating Participants Who Have Tested Positive for Novel Coronavirus 2019 (BOSS-Covid-19). *Clin. Gov.*. https://clinicaltrials.gov/study/NCT04914377.

[227] Li T., Tan Q., Wei C., Zou H., Liu X., Mei Z., Zhang P., Cheng J., Fu J. (2023). Design, Synthesis, and Acute Toxicity Assays for Novel Thymoquinone Derivative TQFL12 in Mice and the Mechanism of Resistance to Toxicity. Molecules.

[228] Allemailem K.S., Alnuqaydan A.M., Almatroudi A., Alrumaihi F., Aljaghwani A., Khalilullah H., Younus H., Khan A., Khan M.A. (2021). Safety and Therapeutic Efficacy of Thymoquinone-Loaded Liposomes against Drug-Sensitive and Drug-Resistant *Acinetobacter baumannii*. Pharmaceutics.

[229] Sanpinit S., Wetchakul P., Chonsut P., Ngamdokmai N., Ahmad A.R., Warinhomhoun S. (2023). Repeated 28-Day Oral Toxicological Study and Gastroprotective Effects of *Nigella sativa* L. Oil (Shuhada) against Ethanol-Induced Gastric Mucosal Injury in Rats. Nutrients.

